# Regulation of antimicrobial activity and xenocoumacins biosynthesis by pH in *Xenorhabdus nematophila*

**DOI:** 10.1186/s12934-017-0813-7

**Published:** 2017-11-15

**Authors:** Shuqi Guo, Shujing Zhang, Xiangling Fang, Qi Liu, Jiangtao Gao, Muhammad Bilal, Yonghong Wang, Xing Zhang

**Affiliations:** 1 Research and Development Center of Biorational Pesticides, Key Laboratory of Plant Protection Resources and Pest Management of Ministry of Education, Northwest A&F University, 22 Xinong Road, Yangling, 712100 Shaanxi China; 2 Shaanxi Research Center of Biopesticide Engineering and Technology, Northwest A&F University, 22 Xinong Road, Yangling, 712100 Shaanxi China; 3 State Key Laboratory of Grassland Agro-ecosystems, College of Pastoral Agriculture Science and Technology, Lanzhou University, Lanzhou, 730020 China; 4 School of Agriculture and Environment, Faculty of Science, The University of Western Australia, 35 Stirling Highway, Crawley, WA 6009 Australia; 5 State Key Laboratory of Microbial Metabolism, School of Life Sciences and Biotechnology, Shanghai Jiao Tong University, Shanghai, 200240 China

**Keywords:** *Xenorhabdus nematophila* YL001, pH, Antibiotic production, Antimicrobial activity, Biological control

## Abstract

**Background:**

Xenocoumacin 1 (Xcn1) and Xenocoumacin 2 (Xcn2) are the main antimicrobial compounds produced by *Xenorhabdus nematophila*. Culture conditions, including pH, had remarkably distinct effects on the antimicrobial activity of *X. nematophila*. However, the regulatory mechanism of pH on the antimicrobial activity and antibiotic production of this bacterium is still lacking.

**Results:**

With the increase of initial pH, the antimicrobial activity of *X. nematophila* YL001 was improved. The levels of Xcn1 and nematophin at pH 8.5 were significantly (*P* < *0.05*) higher than that at pH 5.5 and 7.0. In addition, the expression of *xcnA*-*L*, which are responsible for the production of Xcn1 was increased and the expression of *xcnMN*, which are required for the conversion of Xcn1 to Xcn2 was reduced at pH 8.5. Also, the expression of *ompR* and *cpxR* were decreased at pH 8.5.

**Conclusion:**

The alkaline pH environment was found to be beneficial for the production of Xcn1 and nematophin, which in turn led to high antimicrobial activity of *X. nematophila* at pH 8.5.

**Electronic supplementary material:**

The online version of this article (10.1186/s12934-017-0813-7) contains supplementary material, which is available to authorized users.

## Background

*Xenorhabdus nematophila* is a unique genus of Gram-negative bacteria, belonging to the family Enterobacteriaceae that is mutually associated with an infective dauer juvenile (IJ) insect-pathogenic nematode in the genus *Steinernema* (Steinernematidae) [1]. The primary (I) phase of the bacteria is carried in the intestine of the infective dauer juvenile (IJ) developmental stage of the nematode. The IJ penetrates an insect host and releases the bacteria into the haemocoel. The bacteria rapidly produce various metabolites that can overcome the insect immune system [2], kill the insect, and inhibit the growth of various fungal and bacterial competitors [3–5]. The bacterial symbionts are believed to prevent putrefaction of the insect cadaver and establish conditions that favor the development of both the nematode and bacterial symbionts [6].

Currently, bio-pesticides based on living microbes and their bioactive compounds have been considered as an important component of environmentally-friendly pest management, with the aim to cope with the adverse effects of chemical pesticides on environment and human health. *X. nematophila* has been known to produce several compounds with antimicrobial activity, including indole derivatives, nematophin, benzylideneacetone, xenocoumacins and the major class of non-ribosomally produced secondary metabolites (Xenematides depsipeptides, lysine-rich cyclo PAX-peptides) [7–17]. These metabolites not only have diverse chemical structures, but also a wide range of bioactivities of medicinal and agricultural interest, such as antibiotic, antimycotic, insecticidal, nematicidal, antiulcer, antineoplastic and antiviral. These natural antibiotics provide useful clues in the research and development of drugs and agrochemicals.

Xenocoumacins (Xcn), including Xcn1 and Xcn2, are the antimicrobial compounds produced by *X. nematophila*. Xcn1 exhibits a broad-spectrum antibiotic activity against Gram-positive and Gram-negative bacteria and several fungal species, while Xcn2 is less active against the bacteria and inactive towards the fungal species examined [12]. As Xcn1 is also active against the producer itself, it is detoxified and converted by its own resistance mechanism into the weak antibiotic Xcn2. Xcn1 and Xcn2 are composed of an arginine residue, a leucine residue and four acetate units creating a benzopyran-1-one (isocoumarin) ring structure [12]. The 34-kb biosynthetic gene cluster associated with the production of Xcn contains 14 genes (*xcnA*–*xcnN*), which includes two nonribosomal peptide synthetases (NRPs), three polyketide synthetases (PKSs), and nine accessory genes [18, 19]. The PKSs and NRPs genes *xcnAKFHL* are necessary for Xcn1 synthesis, and the accessory genes *xcnBCDE* are specifically involved in the biosynthesis of the extender unit hydroxymalonyl-ACP [18, 19]. The conversion of Xcn1 into Xcn2 is due to the genes *xcnM* and *xcnN*, which encode proteins homologous to saccharopine dehydrogenases and fatty acid desaturases, respectively [19]. When Xcn2 production is attenuated, an increase in Xcn1 is observed, along with a 20-fold reduction in cell viability. The conversion of Xcn1 to Xcn2 is a resistance mechanism utilized by the bacteria to avoid self-toxicity [18].

Whole-genome sequencing programs highlight the great biosynthetic potential of *X. nematophila* for secondary metabolites, and a large number of biosynthesis gene clusters responsible for the production of secondary metabolites were revealed. Overall, 7.5% of the *X. nematophila* genome contains genes that encode enzymes involved in the synthesis of secondary metabolites, compared with 4.5% for *Streptomyces coelicolor* [20, 21]. The number of the known secondary metabolites was far less than the number of genes encoding biosynthetic enzymes in various bacteria including *X. nematophila* [19]. Many biosynthesis genes remain silent and such cryptic pathways are tightly regulated, and are only activated under specific conditions and only a minority of potential chemical structures is produced under standard laboratory culture conditions. To gain insight to this untapped reservoir of potentially bioactive structures, the strategies to trigger biosynthetic pathways to yield cryptic natural products were developed, these included external cues, co-cultivation and genomic approaches such as genome-mining, epigenetic remodeling, and engineered pathway activation [22, 23]. For example, l-proline, an abundant amino acid in insect hemolymph, contributes to the bacterial proton motive force, up-regulates the production of secondary metabolites and reveals previously cryptic metabolites in *X. nematophila*. By a culture medium designed to mimic the amino acid content of wax moth larval circulatory fluid (a *Galleria mellonella* “hemolymph mimetic medium”, HMM), amicoumacin was found in *X. bovienii* [23].

Culture conditions are critical to the secondary metabolites production of microorganisms. Manipulating culture variables can promote biosynthesis of the secondary metabolite and thus facilitate the discovery of novel natural products [24]. Therefore, it is a prerequisite to design proper culture conditions in an efficient fermentation process. In previous studies, it was found that pH had remarkably effects on the antibiotic activity of *X. nematophila* [25–28]. However, until now, there is no report about the regulatory mechanism of pH on antimicrobial activity and antibiotic production of this bacterium. Thus, it would be interesting to investigate whether the different initial pH will lead to a significantly change in antimicrobial activity and antibiotic production. The objective of this work was to evaluate the difference in antimicrobial activity, antibiotic production and the expression of related gene of antibiotic production of *X. nematophila* under different pH. The antimicrobial activity of the cell-free filtrate, ethyl acetate extract and methanol extract of *X. nematophila* culture under different pH were determined, the metabolomics profiling and the antibiotics production were measured. Also, the expression of *xcn* gene, which is necessary for the synthesis of the major antimicrobial compounds (Xcns) produced by *X. nematophila*, *isn* gene associated with the production of rhabduscin and the response regulator gene, *ompR* and *cpxR* were analyzed. Based on the correlation analysis of the results, the regulation mechanism of pH on antimicrobial activity and antibiotic production were determined.

## Methods

### Symbiotic bacterial strains and culture conditions

*Xenorhabdus nematophila* YL001 was isolated from its nematode symbiont, *Steinernema* sp. YL001 obtained from the soil from Yangling, China [29], and had been identified according to its morphological and molecular characteristics [29, 30]. The entomopathogenic nematode symbiotic bacteria including *X. nematophila* have two phenotypic (previous name: phase) variants, called primary (I) and secondary (II). Only primary (I) exhibits considerable antimicrobial activity [31]. *X. nematophila* YL001 was maintained on nutrient agar (NA) medium and sub-cultured monthly. The primary-secondary switch is spontaneous and in case of *X. nematophila* irreversible. Consequently, the laboratory cultures should be scored after recovering from the 20% glycerol suspension in which they had been stored in the deep freezer. To ensure the predominance of phase I population, refrozen cells were seeded on the surface of NA media supplemented with 0.04% triphenyltetrazolium chloride (w/v) and 0.0025% bromothymol blue (w/v) (NBTA). Phase I is distinguished from II by its adsorption of NBTA to produce a red colony overlaid by dark blue and surrounded by a clear zone after 3–5 days of incubation, and fresh liquid culture were started from single blue (II) colonies [32]. These cultures were scaled up for antibiotics production. Seed culture was prepared by inoculating a loopful of I forms of *X. nematophila* YL001 from a 72 h culture growing on an NBTA plate to a 250-mL flask containing 50 mL fresh NB (NA without agar) medium. The medium was adjusted to a final pH of 7.2 followed by cultivation in darkness at 28 °C on an Erlenmeyer rotary shaker at 180 rpm for 16–24 h, during which time the optical density (600 nm) was approximately 1.50 and 2.00.

The effect of initial pH on the antibiotic activity of the strain was studied using shake flask cultures at different initial pH values. A 250 mL Erlenmeyer flask contained 50 mL medium (WYH) [33] consisting of the following components (g/L): glucose 6.13, peptone 21.29, MgSO_4_·7H_2_O 1.50, (NH_4_)_2_SO_4_ 2.46, KH_2_PO_4_ 0.86, K_2_HPO_4_ 1.11, and Na_2_SO_4_ 1.72. The medium pH was adjusted to 5.5, 6.5, 7.0, 7.5 and 8.5 by adding 1 M NaOH or 1 M HCl. Ten percent (v/v) of the seed culture was used to inoculate the flasks. The culture was incubated on a rotary shaker at 28 °C and 150 rpm for 72 h. Three batches were repeated for each experiment.

### Preparation of cell-free filtrate, ethyl acetate extract bioactive compounds (ethyl acetate extract) and methanol extract bioactive compounds (methanol extract)

Batch cultures were carried out in 250-mL Erlenmeyer flasks containing 50 mL medium with varying initial pH (5.5, 6.5, 7.0, 7.5 and 8.5). Ten percent (v/v) of the seed culture was used to inoculate the flasks. The culture was incubated on a rotary shaker at 28 °C and 150 rpm. After 72 h incubation, the cultures were centrifuged (12,000×*g*, 20 min, 4 °C) to separate the bacterial cells and the cell filtrate was stored at 4 °C until used. The cultures were vigorously extracted with equal volume of ethyl acetate three times, mixed, centrifuged, and the top organic layer was dried for analysis.

Liquid cultures were grown at 150 rpm on a shaker in 250 mL Erlenmeyer flasks containing 50 mL medium and 2% (v/v) of XAD-16 (Sigma-Aldrich). These cultures were inoculated with 10% (v/v) of a 24 h preculture in the same medium without XAD-16. Cultures were harvested after 72 h, and XAD beads were separated from the cells and supernatant by sieving. XAD beads were extracted with MeOH (25 mL), and the MeOH extract was concentrated to dryness on a rotary evaporator. The residue was re-dissolved in MeOH (1.5 mL) for HPLC/MS analysis.

### Assay of antimicrobial activity

The antibacterial activity of cell-free filtrate of *X. nematophila* YL001 culture against six bacteria, including *Bacillus subtilis*, *Bacillus thuringiensis*, *Bacillus cereus*, *Pseudomonas syringae* pv. *actinidiae*, *Xanthomonas campestris* pv. *Oryzae* and *Erwinia carotovora* subsp. *carotovora* were determined using an agar diffusion plate assay [33]. Briefly, 1 mL of medium containing 10^7^–10^8^ colonies of the tested bacterium was applied to an NA plate. After 2 h incubation at room temperature, the supernatants were filtered through a 0.22 μm-pore-size syringe microfilter. The filtrates (60 μL) were placed on 6-mm disc filters (Whatman 3-mm paper; Whatman, Clifton, NJ) and air dried. The dried discs were put on the NA plate and incubated at 28 °C for 48 h to determine the relationship between the size of the zones of inhibition of bacterial growth and the concentration of the antibiotic. Antibacterial activity was expressed as units of activity per mL of the supernatants, where 1 U was defined as a 1.0-mm annular clearing around the antibiotic disk. Wang et al. confirmed the assumption that the changes in the size of the zones of inhibition represented changes in antibiotic concentration [33, 34].

To determine the antimicrobial activity of cell-free filtrate of *X. nematophila* YL001 culture, 13 fungal and oomycete pathogens were selected as listed in Table 1. All the strains used in the present study have been isolated and identified at the Agricultural Culture Collection Institute, Northwest A & F University, China. Except *Magnaporthe grisea* was obtained from rice at Chongqing Province, China. Other strains were all isolated from plant and crops at Shaanxi Province, China. One milliliter cell-free filtrate was mixed with 9 mL potato dextrose agar (PDA) and then poured onto a 9-cm petri dish. Three mycelia PDA plugs (0.4 × 0.4 cm) were cut from the edges of 3 to 5 day colony of each fungi and oomycete (Table 1) growing on PDA at 25 °C and was placed in the center of three plates (one plug in each plate). Thus, for each tested fungi and oomycete, there were three replications (one plate per replication) and the control plates for the comparison of PDA mixed with fermentation medium. The plates were incubated in darkness. After 7 days, colony diameter was measured in two perpendicular directions. The measurements for each plate were terminated when mycelium in one of the control plates reached the edge of the plate. The inhibition rate was determined according to the formula: [(average colony diameter of control − average colony diameter of treatment)/average colony diameter of control] × 100.

**Table 1:** The antimicrobial activity of cell-free filtrates of *X. nematophila* YL001 against different plant pathogens at varying pH values

Fungi and oomycetes	Inhibition rate (%)^1^				
	pH 5.5	pH 6.5	pH 7.0	pH 7.5	pH 8.5
*Alternaria brassicae*	26.86 ± 4.51d	34.69 ± 4.27c	61.78 ± 3.52b	61.36 ± 4.06b	99.28 ± 0.05a
*Alternaria solani*	35.61 ± 5.77d	54.84 ± 0.58c	60.71 ± 0.60c	70.28 ± 0.17b	97.84 ± 0.17a
*Botrytis cinerea*	74.47 ± 0.12b	100.00 ± 0.00a	100.00 ± 0.00a	100.00 ± 0.00a	100.00 ± 0.00a
*Clomerela cinyulate*	23.26 ± 3.05d	73.88 ± 3.05b	76.92 ± 3.01ab	81.22 ± 3.02a	53.67 ± 3.01c
*Curvularia lunata* (Wakker) Boed	41.94 ± 4.01b	70.47 ± 5.01a	69.06 ± 0.29a	68.14 ± 2.01a	71.02 ± 3.56a
*Exserohilum turcicum*	9.77 ± 3.45c	63.51 ± 3.11b	58.91 ± 2.09b	64.08 ± 4.05b	87.64 ± 4.32a
*Fusarium graminearum*	15.96 ± 2.89b	11.82 ± 1.74b	12.84 ± 2.32b	11.63 ± 2.33b	10.84 ± 2.66b
*Fusarium oxysporium*	18.43 ± 4.02cd	25.31 ± 5.20bc	20.15 ± 3.01cd	30.71 ± 4.09ab	37.35 ± 3.01a
*Magnaporthe grisea*	70.93 ± 0.05b	100.00 ± 1.28a	100.00 ± 0.71a	100.0 ± 1.00a	100.00 ± 0.79a
*Physalospora piricola*	5.82 ± 0.09c	36.83 ± 0.04b	100.00 ± 1.36a	96.14 ± 0.29a	100.00 ± 1.82a
*Phytophthora capsici*	13.50 ± 0.90e	39.24 ± 1.24d	72.95 ± 3.00c	85.34 ± 0.34b	91.34 ± 1.00a
*Sclerotinia sclerotiorum*	0.00 ± 0.00d	35.52 ± 5.01b	39.55 ± 3.02b	30.52 ± 2.68c	98.58 ± 0.35a
*Verticillium dahlia*	30.08 ± 4.04d	45.21 ± 5.10c	45.79 ± 4.01c	57.47 ± 4.05b	69.16 ± 4.01a

^1^The inhibition rate of 100 mL/L cell-free filtrate of *X. nematophila* YL001 culture on the mycelial growth of the pathogens tested after 7 days. Data are presented as mean ± SE (n = 6). Different lower case letters followed by the data of each pathogen tested indicate significant differences at *P* = 0.05

To determine the effect of the ethyl acetate extract and methanol extract on the mycelial growth of *B. cinerea*, a stock solution of the ethyl acetate extract and methanol extract (25 mg/mL) of the cell-free filtrate of *X. nematophila* YL001 culture was prepared by dissolving the dried ethyl acetate extract and methanol extract in distilled water. Twofold dilutions were made from the filter-sterilized stock solution. For each dilution, 1 mL was thoroughly mixed with 9 mL PDA and poured into a Petri dish plate. The final concentrations of the ethyl acetate extract and methanol extract in PDA were 0.039, 0.078, 0.156, 0.313, 0.625, 1.25 and 2.5 mg/mL, respectively. Then one mycelial disk (6-mm-diameter) from the edge of 3 to 5 day-old-colony of *B. cinerea* growing on PDA was put onto the center of each plate. For each concentration, there were three replicates (one plate per replicate), and the control plates for comparison were PDA only. The plates were maintained at 25 °C in darkness. The colony diameter of each plate was measured and the inhibitory rate for each concentration on each pathogen was determined after 7 days.

In vivo efficiency of cell-free filtrate, ethyl acetate extract and methanol extract on *B. cinerea* were determined on tomato fruits. Fresh green tomato fruits with uniform size were used. To determine the therapeutic effect, three tomato fruits with the same size were placed at the bottom of each closed plastic container with moisture filter papers at the bottom to maintain high humidity. One mycelia agar disc (4 mm diameter) was taken from the edge of 3- to 5-day colony of *B. cinerea* on PDA and was placed in the middle side of each fruit with mycelia side facing the surface of each fruit. The containers were placed in a climate chamber at 25 °C. After 24 h, the fruits were sprayed immediately with cell-free filtrate and 2.5 mg/mL of ethyl acetate extract. There were three replications (eight fruits per replication) for each treatment. The control for comparisons was sprayed with water, and the 1000 × 80% carbendazim (Bianjing Plant Protection Technology Co., Ltd, suzhou, China) was used as a reference positive chemical control. To determine the protective effect, three tomato fruits were sprayed with each solution as above and kept under the same conditions as above. After 24 h, the fruits were inoculated with *B. cinerea* as described above. After 7 days, lesion diameter was measured in two perpendicular directions. The efficiency rate was determined according to the formula: [(average colony diameter of control − average colony diameter of treatment)/average colony diameter of control] × 100.

### Metabolite analysis of *X. nematophila* YL001 at different pH

The metabolomic profiling of the ethyl acetate extracts from *X. nematophila* YL001 under different initial pH was analyzed by LC–MS. A certain amount dried ethyl acetate extract were re-suspended in 8 mL methanol. LC–MS/MS was performed on a Thermo ion trap LC–MS system (Thermo Scientific, USA) consisting of a surveyor auto sampler, a surveyor MS pump, and an LTQ XLTM linear ion trap mass spectrometer equipped with an ESI source that was operated in the positive mode. The data acquisition software used was Xcalibur 2.1. HPLC separation was carried out using an Agilent ZORBAX Eclipse XDB C_18_ column (250 mm × 4.6 mm, 3.5 µm particle sizes) with an acetonitrile/water gradient at 0.4 mL/min (gradient: 0–2 min, 10% MeCN; 2–20 min, 10–40% MeCN; 20–35 min; 40–100% MeCN, injection volume: 10 μL). The LC–MS/MS conditions were as follows: ESI spray voltage, 4 kV; sheath gas flow rate, 70 arb; auxiliary gas flow rate, 20 arb; capillary voltage, − 38 V; capillary temperature, 350 °C; and tube lens, 95 V. Collision-induced dissociation-MS/MS experiments were performed on the precursor ions selected from MS1 using the selected ion monitoring (SIM) mode: MS1 was performed in the full scan mode (*m/z* 100–1000); MS2 was performed in the SIM mode. The metabolites in ethyl acetate extract at different pH were identified based on LC–MS/MS. Relative amount of metabolites = [the peak area of extracted ion chromatograms (EIC) of metabolites at different pH/the peak area of extracted ion chromatograms (EIC) of metabolites at pH 8.5] × 100. The relative amount of metabolites at pH 8.5 refers to 100.

The analysis of the production of Xcns was performed by a liquid chromatography–tandem mass spectrometry (LC–MS/MS, API 2000, AB Sciex, USA). A certain amount dried methanol extract was re-suspended in 8.0 mL methanol for analysis. For the determination of Xcn1 and Xcn2, the molecular ions *m/z* [M+H]^+^ 466.3 and 407.3 were quantified, respectively, using the API2000 LC–MS system equipped with an ESI source that was operated in the positive mode. HPLC separation was carried out using an Agilent C_18_ column (150 mm × 4.6 mm, 5 µm) with an acetonitrile/water gradient (+ 0.1% formic acid) at 0.4 mL/min (gradient: 0–22 min, 5–95% MeCN; 22–24 min, 95–5% MeCN; 24–30 min, 5% MeCN; 40–45 min, 100–10% MeCN; 45–55 min, 10% MeCN, injection volume: 10 μL). The LC–MS/MS conditions: auxiliary gas flow rate, 20 arb; sheath gas flow rate, 70 arb; capillary voltage, − 38 V; ESI spray voltage, 4 kV; and tube lens, 95 V; capillary temperature, 350 °C. The full scan mode (*m/z* 100–1000) was applied for MS1. MS/MS experiments were performed on the precursor ions selected from MS1 using the selected ion monitoring (SIM) mode. The relative amount of Xcns was calculated by the following equation: the relative amount of Xcns = [the peak area of extracted ion chromatograms (EIC) of Xcns at different pH/the peak area of extracted ion chromatograms (EIC) of Xcns at pH 8.5] × 100, the relative amount of Xcns at pH 8.5 was referred to 100. All analyses were performed in triplicate.

### RT-PCR and qRT-PCR analysis

Standard conditions for isolating total RNA from cells grown in WYH medium were as follows: *X. nematophila* YL001 strains were initially grown overnight in LB, Ten percent (v/v) of the seed culture was used to inoculated into WYH media with different initial pH in a 250 mL flask and incubated to desired cell density. For RT-PCR analysis, exponentially growing cells were used to extract total RNA use total RNA isolation system (Promega). RT-PCR was performed using RevertAiD First Strand cDNA Synthesis Kit (Thermo). The RT-PCR reaction (25 μL) contained the following components: 300 ng of total RNA, 20 pmol each of forward and reverse primer and 200 units of Revert Aid M-MuLV Reverse Transcriptas. The cDNA synthesis was conducted at 42 °C for 60 min. The following cycle condition was used for PCR reaction: 30 s at 94 °C, 30 s at 55 °C and 60 s at 72 °C for extension. Annealing temperature varied depending on which primer was used. For RT-PCR analysis, 26–30 cycles of PCR reaction was used for all *xcn* genes. 16S rDNA was used as the internal control gene to confirm that equal amounts of total RNA was used in each reaction. Quantitative RT-PCR was performed using PrimeScript™ RT reagent Kit with gDNA Eraser (TaKaRa) and SYBR^®^ Premix ExTaq™ II (TliRNaseH plus) (TaKaRa) and method using Thermal Cycler Dice Real Time System. Equal amounts of DNase-treated total RNA (300 ng) were used to generate cDNA. The resultant cDNA was diluted ten times with sterilized distilled water. The diluted cDNA (2.0 μL) was subsequently used in 25 μL qRT-PCR reaction, which was carried out in triplicate on cDNA Iq™5. Sequences of primers used in RT-PCR and qRT-PCR are shown (Additional file 1: Table S1). Cycle threshold (Ct) results and melting curve for each sample were generated by Opticon MonitorTM software (Version 1.0). The fold changes in the amount of *xcnA*, *xcnM* and *xcnN* transcripts (target genes) relative to the *recA* transcripts (control gene) were determined by the following equation: fold change = 2^−Δ(ΔCt)^; ΔCt = Ct_target_ − Ct_control_; and Δ(ΔCt) = ΔCt_treatment1_ − ΔCt_treatment2_; final mean values of Δ(ΔCt) and fold changes were obtained from three independent RNA samples.

### Data analyses

Data analyses were conducted using the SPSS statistical package (version 18.0 for windows; SPSS Inc., Chicago, IL) and Origin Pro 9.1. Analyses of variance were carried out to determine the effects of different treatments at different initial pH. Subsequent multiple range comparisons between means were determined based on least significant differences (l.s.d) at *P* < 0.05.

## Results

### In vitro antimicrobial activity of the cell-free filtrate, ethyl acetate extract and methanol extract

The cell-free filtrate of *X. nematophila* YL001 under different initial pH showed an extensive antimicrobial activity against the fungal and oomycete pathogens tested, with a range of 70.93–100% for *M. grisea*, 35.61–97.84% for *A. solani*, 26.86–99.28% for *A. brassicae*, 74.47–100.00% for *B. cinerea*, 5.82–100.00% for *P. piricola* and 0.00–98.58% for *S. sclerotiorum* (Table 1). In particular, the cell-free filtrates of *X. nematophila* YL001 under initial pH 8.5 exhibited an inhibitory effect greater than 90% on *A. solani*, *A. brassicae*, *B. cinerea*, *M. grisea*, *P. piricola*, *P. capsici* and *S. sclerotiorum*. Among these, *B. cinerea* was selected as the pathogens for subsequent studies.

**Fig. 1:**
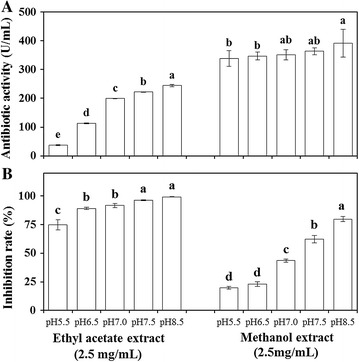
Inhibitory effect of ethyl acetate extract and methanol extract of the cell-free filtrate of *X. neamatophila* YL001 at different initial pH on *B. subtilis* (**A**) and *B. cinerea* (**B**). The inhibitory effect of *X. nematophila* YL001 culture on *B. subtilis* is presented as antibiotic activity which was expressed as units of activity per mL of the cell-free filtrate generated as described in “Methods”. The inhibitory effect of *X. nematophila* YL001 culture on *B. cinerea* is presented as inhibition rate which was calculated described in “Methods”. Data are presented as the averages ± the standard deviations for six replicates. Different lower case letters above the bars indicate significant differences at *P* = 0.05

The ethyl acetate extract and methanol extract of the cell-free filtrate of *X. nematophila* YL001 under different initial pH exhibited inhibitory effect on the mycelial growth of *B. cinerea*, with a range of 74.71–99.23% and 19.62–79.89% (Fig. 1B), respectively. Notably, the ethyl acetate extract and methanol extract exhibited strong inhibition effects on the mycelial growth of *B. cinerea* under initial pH 8.5, with an inhibition rate of 99.23 and 79.89%, respectively. In addition, at pH 8.5, the concentration of ethyl acetate extract and methanol extract from 0.039 to 2.5 mg/mL all showed inhibitory effect on the mycelial growth of *B. cinerea*, and there was a liner relationship between the concentration of ethyl acetate extract, methanol extract and inhibitory rate on the mycelial growth of *B. cinerea* (Table 2). The ethyl acetate extract showed stronger inhibitory effect on *B. cinerea* than the methanol extract, with an EC_50_ of 1.294 and 1.340 mg/mL, respectively (Table 2).

**Table 2:** Effect of ethyl acetate extract and methanol extract of the cell-free filtrate of *X. neamatophila* YL001 culture at pH 8.5 on mycelial growth of *B. cinerea*

	Regression equation^a^	EC_50_ (mg/mL)^b^	*r* ^c^	95% confidence interval
Ethyl acetate extract	*y* = − 0.185 + 1.653*x*	1.294	0.9439	1.000–1.930
Methanol extract	*y* = 0.462 + 0.299*x*	1.340	0.9706	0.628–3.032

^a^The liner relationship between log10-transformed concentration of ethyl acetate extract and methanol extract (*x*) and probit-transformed inhibitory rate on the mycelia growth of *B. cinerea* (*y*)

^b^EC_50_, 50% effective concentrations

^c^Coefficient of correlation

The cell-free filtrate of *X. nematophila* YL001 under different initial pH also showed an extensive inhibitory effect on the bacterial pathogens tested, with a range of 96.0–210.0 U/mL for *B. subtilis* and 13.0–132.0 U/mL for *P. syringae* (Table 3). The ethyl acetate extract and methanol extract of the cell-free filtrate of *X. nematophila* YL001 under different initial pH exhibited a potent inhibition effect on *B. subtilis*, with a range of 37.50–244.17 and 339.0–391.0 U/mL (Fig. 1A), respectively. In particular, under initial pH 8.5, the ethyl acetate extract and methanol extract exhibited strong antibiotic activity on *B. subtilis*, with an antibiotic activity 244.17 and 391.0 U/mL, respectively.

**Table 3:** The inhibitory effect of cell-free filtrates of *X. nematophila* YL001 on the selected bacterial pathogens at varying initial pH

Bacteria	Antibiotic activity (U/mL)^1^				
	pH 5.5	pH 6.5	pH 7.0	pH 7.5	pH 8.5
*Bacillus subtilis*	96.0 ± 17.0c	194.0 ± 6.0b	205.0 ± 13.0a	205.0 ± 6.0a	210.0 ± 29.0a
*Bacillus thuringiensis*	8.0 ± 0.6e	77.0 ± 1.2d	73.0 ± 1.2c	81.0 ± 6.0b	92.0 ± 1.2a
*Bacillus cereus*	17.0 ± 1.2d	44.0 ± 6.0c	84.0 ± 6.0b	84.0 ± 3.0b	95.0 ± 1.7a
*Pseudomonas syringae* pv. *actinidiae*	13.0 ± 6.0d	84.0 ± 2.3c	107.0 ± 12.0b	128.0 ± 2.9a	132.0 ± 6.0a
*Xanthomonas campestris* pv. *oryzae*	15.0 ± 1.2c	26.0 ± 1.2b	24.0 ± 6b	32.3 ± 2ab	34.0 ± 3.0a
*Erwinia carotorora* subsp. *carotovora*	18.0 ± 0.6b	9.0 ± 2.9c	13.0 ± 0.6b	20.0 ± 0.6a	22.7 ± 1.5a

^1^Antibiotic activity of *X. nematophila* YL001 culture on the bacterial pathogens tested was expressed as units of activity per mL of the cell-free filtrate. Data are presented as mean ± SE (n = 6). Different lower case letters followed by the data of each pathogen tested indicate significant differences at *P* = 0.05

### In vivo efficiency of the cell-free filtrate, ethyl acetate extract and methanol extract on tomato fruits infected with *B. cinerea*

**Fig. 2:**
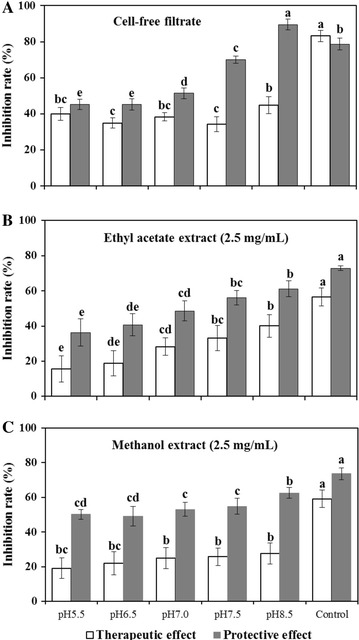
Effect of *X. neamatophila* YL001 at different initial pH on grey mold of detached tomato fruits caused by *B. cinerea*. **A** The therapeutic and protective of ell-free filtrate. Data are presented as the averages ± the standard deviations for six replicates. **B** The therapeutic and protective of ethyl acetate extract at 2.5 mg/mL. Data are presented as the averages ± the standard deviations for six replicates. **C** The therapeutic and protective of methanol extract at 2.5 mg/mL. Data are presented as the averages ± the standard deviations for six replicates. The chemical control is 80% carbendazim (1.0 mg/mL). Different capital letters above the bars indicate significant differences at *P* = 0.05

There was a significant efficiency (*P* < 0.05) of treatments (the cell-free filtrate, ethyl acetate extract and methanol-extract) under different initial pH on detached tomato fruits infected with *B. cinerea*. The therapeutic and protective effects of treatments under different initial pH on *B. cinerea* were improved with the increase of initial pH. The protective efficacy was higher than therapeutic efficacy at each treatment (Fig. 2). At pH 8.5, the therapeutic effect of cell-free filtrate (Fig. 2A), ethyl acetate extract (Fig. 2B) and methanol-extract (Fig. 2C) was 44.81, 39.94 and 27.55%, respectively; the protective effect of cell-free filtrate, ethyl acetate extract and methanol-extract was 89.54, 61.22 and 62.57%, respectively, and there was a significant difference (*P* < 0.05) between therapeutic effect and protective effect. At pH 8.5, the protective effect of cell-free filtrate (89.54%) was higher than that of chemical control (78.69%).

### Effect of pH on gene expression

**Fig. 3:**
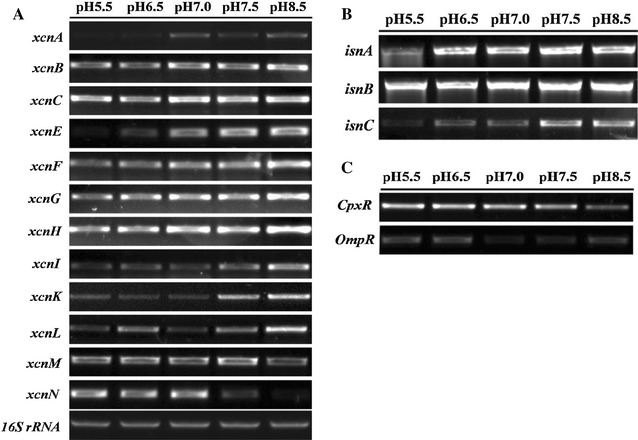
RT-PCR analysis of different genes of *X. nematophila* YL001 under different initial pH. Total RNA was obtained from *X. nematophila* YL001 during exponential growth in WYH medium mentioned in “Methods”. **A** *xcn* genes, **B** *isn* genes, **C** *cpxR* and *ompR* gene

**Fig. 4:**
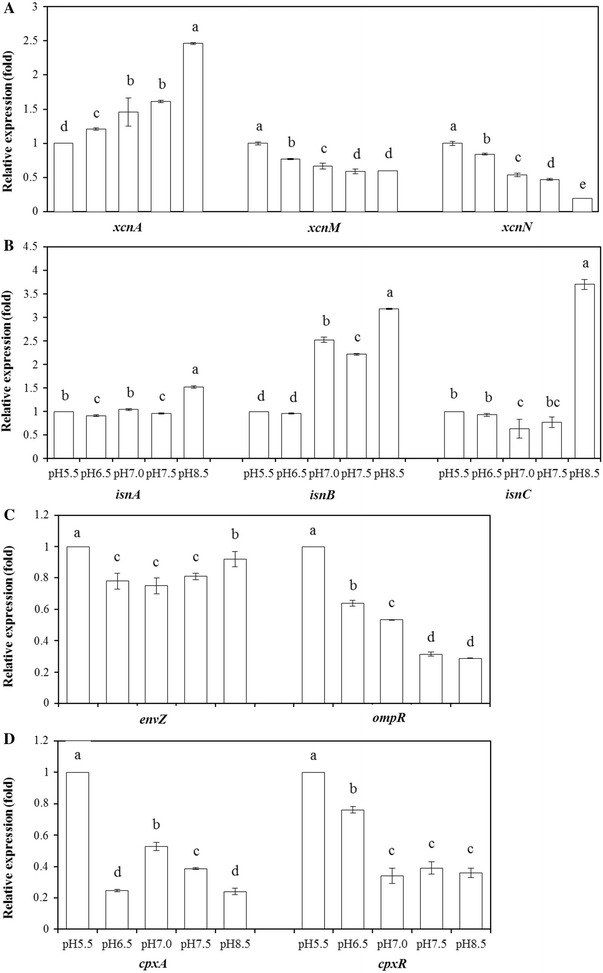
Quantitative RT-PCR analyses of different genes of *X. nematophila* YL001 under different initial pH. Total RNA was obtained from *X. nematophila* YL001 during exponential growth in WYH medium mentioned in “Methods”. **A** *xcnA*, *xcnM* and *xcnN* gene, **B** *isn* genes, **C** *envZ* and *ompR* gene, **D** *cpxA* and *cpxR* gene. Data are presented as the averages ± the standard deviations for six replicates. Different capital letters above the bars indicate significant differences at *P* = 0.05

Xcn are the antimicrobial compounds produced by *X. nematophila*. The biosynthetic gene cluster associated with the production of Xcn contains 14 genes (*xcnA*–*xcnN*). To evaluate the contribution of pH in *xcn* genes expression, we determined the expression of the main genes required for the Xcns by RT-PCR. RT-PCR analysis revealed that the level of mRNA expression for *xcnA*-*L* was increased with the increase of the initial pH, while the expression of *xcnM* and *xcnN* were decreased (Fig. 3a). These findings were supported by qRT-PCR analysis that *xcnA* expression increased 2.50-fold, while *xcnM* and *xcnN* expression decreased 0.40- and 0.81-fold at pH 8.5, compared with that at pH 5.5 (Fig. 4A).

Rhabduscin takes an important part in the melanization pathway of the insect’s immune system [35]. The rhabduscin gene cluster (*isn*) has been identified in genomes from both *Xenorhabdus* and *Photorhabdus* [21, 36]. RT-PCR analysis revealed that the expression level of *isnA*, *isnB* and *isnC* were increased with the increase of the initial pH (Fig. 3b), this was also supported by qRT-PCR analysis that *isnA*, *isnB* and *isnC* expression were 0.53-, 2.18- and 2.71-fold higher at pH 8.5 relative to pH 5.5, respectively (Fig. 4B).

CpxR and OmpR are response regulators involved in the regulation of bacterial pathogenicity and mutualism, which negatively regulate antibiotic activities of *X. nematophila*. RT-PCR analysis showed that *cpxR* and *ompR* expressed at lower levels with the increase of the initial pH from 5.5 to 8.5 (Fig. 3c). The result was also supported by qRT-PCR analysis that *cpxR* and *ompR* expression decreased 0.64- and 0.71-folds at pH 8.5, compared with that at pH 5.5 (Fig. 4C, D).

### Metabolites analysis of *X. nematophila* at different pH

**Fig. 5:**
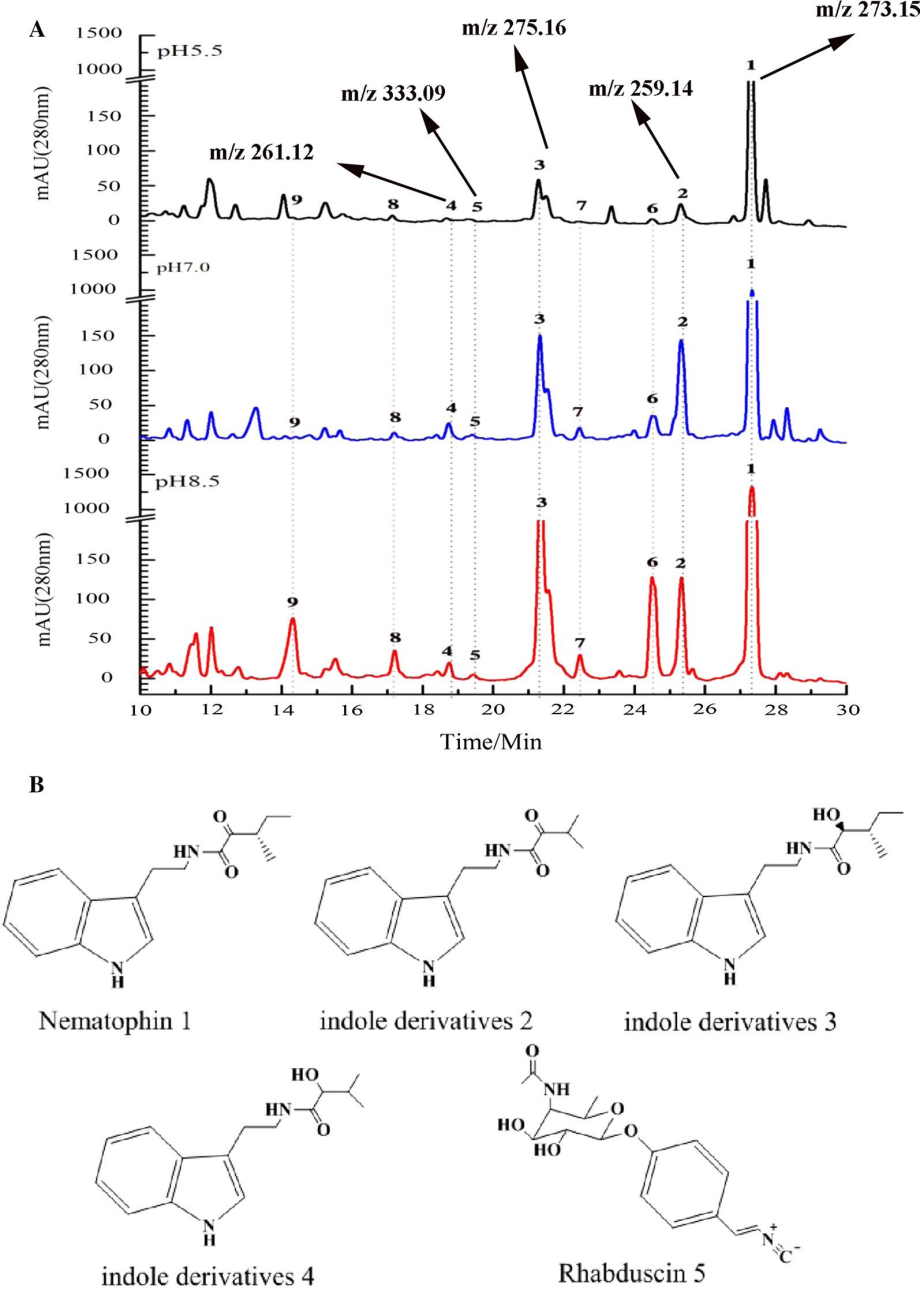
HPLC–MS analysis of metabolite profiling of the ethyl acetate extract of cell-free filtrate of *X. neamatophila* YL001 under different initial pH. **A** Comparison of base peak chromatograms of *X. nematophila* YL001 at pH 5.5 (up), 7.0 (middle) and 8.5 (down). **B** LC–MS/MS analysis detecting the metabolites in the ethyl acetate extract of cell-free filtrate of *X. nematophila* YL001 (Additional file 1: Figures S2–S4). Peak 1, nematophin, *m/z* 273.15 [M+H]^+^; peak 2, Indole derives, *m/z* 259.14 [M+H]^+^; peak 3, Indole derives, *m/z* 275.16 [M+H]^+^; peak 4, indole derives, *m/z* 261.12 [M+H]^+^; peak 5, rhabduscin, *m/z* 333.00 [M+H]^+^

The chemical compositions of ethyl acetate extracts and methanol extract were by analyzed LC–MS/MS. The metabolomic profiling of ethyl acetate extracts from *X. nematophila* YL001 under different initial pH had significant difference (Fig. 5a). Five compounds (Fig. 5b), nematophin (1), indole derivatives (2–4) and rhabduscin (5), were well identified by LC–MS/MS (Additional file 1: Table S2; Figures S2–S4). Under pH 8.5, this bioactive small molecules, nematophin, rhabduscin and indole derivatives, were up-regulated compared with that at pH 7.0 and 5.5 (Fig. 5a, peaks 1, 2, 3, 4 and 5), and some previously cryptic metabolites evident in the differential profiles (Fig. 5a, peaks 7, 8, and 9). Rhabduscin and nematophin production were 24.57- and 2.02-folds higher at pH 8.5 relative to that at pH 5.5, respectively (Table 4).

**Table 4:** The relative amount of rhabduscin, nematophin and indole derivatives in ethyl acetate extract of the cell-free filtrate of *X. neamatophila* YL001 at pH 5.5, 7.0 and 8.5

Treatment	Relative amount of metabolites^a^				
	Nematophin	Indole derivative 2	Indole derivative 3	Indole derivative 4	Rhabduscin
pH 8.5	100.00	100.00	100.00	100.00	100.00
pH 7.0	63.51	93.90	63.91	91.98	10.46
pH 5.5	49.36	26.48	48.81	25.55	4.07

^a^The metabolites in ethyl acetate extract of the cell-free filtrate of *X. neamatophila* YL001 at different pH were identified based on LC-MS/MS in Additional file 1: Figures S2–S4, and illustrated in Fig. 5. The chromatogram peak of identified metabolites at different pH refers to Fig. 5. Relative amount of metabolites = [the peak area of extracted ion chromatograms (EIC) of metabolites at different pH/the peak area of extracted ion chromatograms (EIC) of metabolites at pH 8.5] × 100. The relative amount of metabolites at pH 8.5 refers to 100

In order to further evaluate the effect of pH on the production of metabolites, we characterized several compounds from *X. nematophila*. After purification of the metabolites, NMR and GC–MS experiments were performed, identifying one of them as nematophin (Additional file 1: Table S3; Figures S6–S8). HPLC analysis shown that the concentration of nematophin produced over 72 h by *X. nematophila* Yl001 at pH 5.5, 7.0 and 8.5 were 33.20, 146.42 and 228.12 μg/mL, respectively (Additional file 1: Figure S5).

The metabolomic profiling of methanol extract from *X. nematophila* YL001 under different initial pH had significant difference. The production of the water-soluble peptide antimicrobial compound Xcn1, the major antibiotics produced by *X. nematophila*, were 16.36- and 2.49-fold higher at pH 8.5 relative to that at pH 5.5 and pH 7.0 (Table 5, Additional file 1: Figures S9–S11).

**Table 5:** The relative amount of xenocoumacins (Xcn1 and Xcn2) in methanol extract of the cell-free filtrate of *X. neamatophila* YL001 at pH 5.5, 7.0 and 8.5

Treatment	Relative amount of Xcns^1^	
	Xcn1	Xcn2
pH8.5	100.000a	100.000a
pH7.0	40.171b	51.206b
pH5.5	6.114c	10.080c

^1^The relative amount of Xcn1 (the molecular ion [M+H]^+^, 466 *m/z*) and Xcn2 (the molecular ion [M+H]^+^, 407 *m/z*) in methanol extract of the cell-free filtrate of *X. neamatophila* YL001 at different pH were determinated by ESI-HPLC–MS (See Additional file 1: Figures S9–S11). Relative amount of Xcns = [the peak area of extracted ion chromatograms (EIC) of Xcns at different pH/the peak area of extracted ion chromatograms (EIC) of Xcns at pH 8.5] × 100. The relative amount of Xcns at pH 8.5 refers to 100. Different lower case letters followed by the data of Xcn1 and Xcn2 at different pH indicate significant differences at *P* = 0.05

## Discussion

In vitro and in vivo, analysis of the antimicrobial activity of *X. nematophila* YL001 at different initial pH indicated that the alkaline pH conditions were beneficial to antibiotic production. Previous studies have reported the variation in antimicrobial activities of different *Xenorhabdus* spp. strains to fungal pathogens at different pH [25, 26]. The higher and lower initial pH value (e.g., pH 4.0 and 1.0) were not beneficial to cell growth and antibiotic production of *X. nematophila* YL001, and the influence of pH on cell growth was not significant as compared to that of antibiotic production within the range of 5.5–8.5 [28]. Similar results appeared in the other work [25, 26] in which it was reported that the antibiotic production by *X. nematophila* BJ and *Xenorhabdus* sp. D43 were affected by the initial pH within the range of 4.5–8.5 and optimal initial pH for cell growth and antibiotic production occurred at 6.0–8.0. These results could also be validated by the profiles of pH variation of the culture broth of *X. nematophila* YL001 at different initial pH. At initial pH 5.5, 7.0 and 8.5 with the use of glucose as sole carbon source, the pH of the culture broth has a natural tendency to change along with the bioprocess, the culture broth pH of YL001 decline from 5.5, 7.0 and 8.5 to pH 5.08, 6.1 and 6.45 during the first few hours of growth, and then increased throughout the course of the experiment reaching 8.4, 8.79 and 8.57 at 72 h, respectively (data not shown). Similar results were obtained in the reports of Yang et al. [25, 26], the culture broth pH of *X. nematophila* BJ varied from 6.5 to 8.5.

In our previous studies, it was found that at initial pH 7.0, the pH of the fermentation broth of *X. nematophila* YL001 varied between 5.90 and 9.75 [28]. Low and high pH values (e.g., constant pH 4.5 and 9.5) were disadvantageous to cell growth and antibiotic production. The result indicated that the pH variation values were near the extreme values at initial pH 7.0 [28]. In this study, the pH variation values proceeds within the acceptable ranges at initial pH 5.5, 7.0 and 8.5.

Manipulating nutritional or environmental factors can promote secondary metabolite biosynthesis of microorganisms enabling the discovery of new natural products. Molecules present in the insect hemolymph can selectively stimulate the secondary metabolic pathways in the *Photorhabdus* and *Xenorhabdus* [17, 36, 37]. Recently, Crawford group designed a culture medium to mimic the amino acid content of wax moth larval circulatory fluid (a *Galleria mellonella* “hemolymph mimetic medium”), which stimulated *X. bovienii* to produce amicoumacins [23]. Due to the structural-relationship between amicoumacins and xenocoumacins (Xcns), this novel nutritional stimulation adjustment approach may offer a new route to regulate xenocoumacins and other antibiotics production in *X. nematophila*. In present study, the influence of an external environmental stimulus (pH shock) on the production of antimicrobial compounds in *X. nematophila* was explored. Similar to nutrient stimulation signals, pH shock not only enhanced the levels of some antibiotics but also induced the production of several cryptic metabolites by activating the related secondary metabolic pathways (Fig. 5). In this study, the increased antimicrobial activity of *X. nematophila* YL001 under pH 8.5 (Figs. 1, 2) suggested that antibiotics might be produced at elevated levels or the cryptic biosynthetic pathway of secondary metabolites were induced in this condition. To address this possibility, the metabolomics profiling of organic extracts from *X. nematophila* YL001 under different initial pH and the antibiotics production were analyzed by HPLC–MS [17, 18]. The previously described molecules exhibited a range of biological activities, and their regulation by pH underscores the connection between recognition of the pH of the environment and subsequent bacterial metabolic adjustment. The pH of the environment significantly altered indole-containing metabolites (structure 1–4), rhabduscin (structure 5) and xenocoumacins (Xcn). At pH 8.5, the production of nematophin (structure 1) and its reduction product (structure 3) increased, which illustrates that the pH of the environment regulates a general up-regulation rather than a metabolic shift. Nematophin production was 1.57- and 2.02-folds higher at pH 8.5 relative to that at pH 7.0 and 5.5, respectively (Table 4). These may explain why the antibacterial and antifungal activity of ethyl acetate extracts against *B. subtilis* and *B. cinerea* were 5.51- and 0.33-folds higher at pH 8.5 than pH 5.5, respectively (Fig. 1). Also, the production of the water-soluble peptide antimicrobial compound Xcn1, the major antibiotics produced by *X. nematophila*, was 16.36-fold higher at pH 8.5 relative to that at pH 5.5. The result may explain why the antibacterial and antifungal activity of methanol extracts against *B. subtilis* and *B. cinerea* were 1.188- and 0.343-folds higher at pH 8.5 relative to that at pH 5.5, respectively (Fig. 1). Meanwhile, alkaline pH (pH 8.5) could induce the production of previously cryptic metabolites (Fig. 5, peaks 7, 8, and 9). However, the biological activities of these induced cryptic metabolites need further validated by purification and characterization. Taken together, the increased antimicrobial activity of *X. nematophila* YL001 under pH 8.5 was likely due to the combined effect of elevated Xcn1 and nematophin or the cryptic antibiotics induced in this condition. Differential metabolomics profiling with relevant environment factors provides a system-wide approach to coupling small-molecule metabolites to the bacteria’s physiological state.

In present study, under pH 8.5 the transcription levels of *xcnA*-*L* genes, which were responsible for Xcn1 synthesis, were significantly higher than that at other pH condition, but *xcnM* and *xcnN*, which responsible for the conversion of Xcn1 into Xcn2, were expressed at lower levels. These finding were supported by the result of LC–MS analysis of methanol extracts showing that Xcn1 increased 16.36-fold at pH 8.5 relative to that at pH 5.5. Thus, the 16.36-fold increase in Xcn1 production at pH 8.5 was likely due to the combined effect of elevated expression of the *xcnA*-*L* genes and reduced *xcnMN* expression. Also, under pH 8.5, the transcription levels of *ompR* and *cpxR* gene, encoding the response regulator (OmpR and CpxR) of CpxRA and EnvZ/OmpR two-component signal transduction system, were significantly lower than that at other pH condition. In *X. nematophila* CpxRA and EnvZ/OmpR are used to sense environmental stresses and transduce the information inside the cells to regulate the pathogenic and mutualistic interactions of *X. nematophila* for the adaptation of the nematode and insect environments [38–40]. Crawford et al. [17] found that increased osmotic stress generally stimulated metabolite production in *X. nematophila* cultures, and l-proline provided further stimulation. When *X. nematophila* multiply in the insect hemolymph, which has long been known to contain high solute (e.g., l-proline, sugars, polyols and derivatives) and inorganic salt concentrations to manage water losses due to environmental osmotic stresses [35], EnvZ senses high osmolarity of hemolymph and activates OmpR to coordinately repress flagella synthesis, exoenzyme and antibiotic production in *X. nematophila* by negatively regulating the *flhDC* operon [38]. Also, OmpR was found to negatively regulate *xcnA*-*L* genes expression and positively regulate *xcnMN* expression [18, 19]. In *ompR* mutant strain Xcn1 levels and expression of *xcnA*-*L* were increased while Xcn2 levels and *xcnMN* expression were reduced [19]. Our findings further validated the notion of Park et al. (2009) that OmpR negatively regulate the production of Xcn1 and positively regulate the production of Xcn2.

Upon recognition of the signal of the change in pH, CpxA phosphorylates CpxR, activating this response regulator, and regulate the expression of its regulon which include CpxR [41]. In this study, *cpxR* expressed at lower levels with the increase of the initial pH from 5.5 to 8.5, indicating that at pH 8.5 CpxR repress its own expression. Also, it is interesting to find that at pH 8.5, Xcn1 levels and *xcnA*-*L* expression were increased while *xcnMN* expression was decreased. As OmpR, CpxR was found to negatively regulate *xcnA*-*L* gene expression, and positively regulate *xcnMN* expression [42]. In *cpxR* mutant strain Xcn1 levels and expression of *xcnA*-*L* were increased while Xcn2 levels and *xcnMN* expression were reduced [42]. A similar regulatory overlap appears to occur in *E. coli* the CpxR regulon overlaps the regulons of the two-component regulator OmpR [43, 44]. The uniform effects of *X. nematophila* CpxR and OmpR suggest that these factors directly regulate similar genes in uniform fashions or that one of these regulators positively regulates the other, indirectly affecting downstream genes. The conclusion was supported by the results that in the *cpxR* mutant the expression of *ompR* decreased compared to the wild strain [42]. Together, these findings support a model in which at pH 8.5, CpxA senses high pH, activates CpxR, and CpxR repress its own expression, and CpxR either directly or indirectly positively regulates *ompR* expression and *ompR* expression was decreased, however OmpR negatively regulate the production of Xcn1 and positively regulate the production of Xcn2. So, at pH 8.5 Xcn1 was shown to be produced at high levels and *X. nematophila* YL001 exhibited high antimicrobial activity.

## Conclusions

In summary, with the increase of initial pH, the antimicrobial activity of *X. nematophila* YL001 increased. At pH 8.5, nematophin and Xcn, the major antibiotics produced by *X. nematophila*, were increased remarkably. So, the increased antimicrobial activity might be even synergistic effects of these compounds acting together, but we did not exclude to the production of a second and probably still unknown compound at pH 8.5. Further work will be focused to isolate and characterize the antimicrobial compounds at pH 8.5 to clarify the problem.

## Additional file

**Additional file 1: Table S1.** Primers used in this study. **Table S2.** Information of the identified metabolites in ethyl acetate extract of *X. nematophila* YL001 based on LC–MS/MS. **Table S3.** ^1^H and ^13^C data for nematophin in CDCl_3_. Chemical shifts (δ) in ppm. **Figure S1.** Inhibitory effect of ethyl acetate extract and methanol extract of the cell-free filtrate of *X. neamatophila* YL001 at different initial pH on *B. subtilis* (A) and *B. cinerea* (B). **Figure S2.** HPLC–MS TIC of the identified metabolites in ethyl acetate extract of *X. nematophila* YL001 at pH 8.5. **Figure S3.** HPLC–MS TIC of the identified metabolites in ethyl acetate extract of *X. nematophila* YL001 at pH 7.0. **Figure S4.** HPLC–MS TIC of the identified metabolites in ethyl acetate extract of *X. nematophila* YL001 at pH 5.5. **Figure S5.** HPLC analysis of nematophin produced by *X. nematophila* YL001 at pH 5.5, 7.0 and 8.5. **Figure S6.** ^1^H-NMR spectra (500 MHz, CDCl_3_) of nematophin. **Figure S7.** ^13^C-NMR spectra (125 MHz, CDCl_3_) of nematophin. **Figure S8.** GC–MS analysis of nematophin. **Figure S9.** HPLC–MS analysis of Xcn1 and Xcn2 produced by *X. nematophila* YL001 at pH 8.5. **Figure S10.** HPLC–MS analysis of Xcn1 and Xcn2 produced by *X. nematophila* YL001 at pH 7.0. **Figure S11.** HPLC–MS analysis of Xcn1 and Xcn2 produced by *X. nematophila* YL001 at pH 5.5.
