# Supplementary material for: Regulation of antimicrobial activity and xenocoumacins biosynthesis by pH in Xenorhabdus nematophila
Source: Microb Cell Fact. 2017 Nov 15;16:203. doi: 10.1186/s12934-017-0813-7 (PMC5688692; doi:10.1186/s12934-017-0813-7)
Supplement: Supplementary file 1 — Additional file 1: Table S1. Primers used in this study. Table S2. Information of the identified metabolites in ethyl acetate extract of X. nematophila YL001 based on LC–MS/MS. Table S3. 1H and 13C data for nematophin in CDCl3. Chemical shifts (δ) in ppm. Figure S1. Inhibitory effect of ethyl acetate extract and methanol extract of the cell-free filtrate of X. neamatophila YL001 at different initial pH on B. subtilis (A) and B. cinerea (B). Figure S2. HPLC–MS TIC of the identified metabolites in ethyl acetate extract of X. nematophila YL001 at pH 8.5. Figure S3. HPLC–MS TIC of the identified metabolites in ethyl acetate extract of X. nematophila YL001 at pH 7.0. Figure S4. HPLC–MS TIC of the identified metabolites in ethyl acetate extract of X. nematophila YL001 at pH 5.5. Figure S5. HPLC analysis of nematophin produced by X. nematophila YL001 at pH 5.5, 7.0 and 8.5. Figure S6. 1H-NMR spectra (500 MHz, CDCl3) of nematophin. Figure S7. 13C-NMR spectra (125 MHz, CDCl3) of nematophin. Figure S8. GC–MS analysis of nematophin. Figure S9. HPLC–MS analysis of Xcn1 and Xcn2 produced by X. nematophila YL001 at pH 8.5. Figure S10. HPLC–MS analysis of Xcn1 and Xcn2 produced by X. nematophila YL001 at pH 7.0. Figure S11. HPLC–MS analysis of Xcn1 and Xcn2 produced by X. nematophila YL001 at pH 5.5. [file 12934_2017_813_MOESM1_ESM.doc]

**Supplementary Information**

**Regulation of antimicrobial activity and xenocoumacins biosynthesis by pH in *Xenorhabdus nematophila***

Shuqi Guo^1,5#^, Shujing Zhang^1#^, Xiangling Fang^3,4^, Qi Liu^1^, Jiangtao Gao^1^, Muhammad Bilal^5^, Yonghong Wang^1,2*^, Xing Zhang^1,2^

^1^Research and Development Center of Biorational Pesticides, Key Laboratory of Plant Protection Resources and Pest Management of Ministry of Education, Northwest A&F University, 22 Xinong Road, Yangling, Shaanxi 712100, China

^2^Shaanxi Research Center of Biopesticide Engineering and Technology, Northwest A&F University, 22 Xinong Road, Yangling, Shaanxi 712100, China

^3^State Key Laboratory of Grassland Agro-ecosystems, College of Pastoral Agriculture Science and Technology, Lanzhou University, Lanzhou 730020, China

^4^School of Agriculture and Environment, Faculty of Science, The University of Western Australia, 35 Stirling Highway, Crawley, WA 6009, Australia

^5^State Key Laboratory of Microbial Metabolism, School of Life Sciences and Biotechnology, Shanghai Jiao Tong University, Shanghai, 200240, China

**^#^**These authors contributed equally to this work.

*Correspondence: Y.H. Wang ([yhwang@nwsuaf.edu.cn](mailto:yhwang@nwsuaf.edu.cn))

**Supplemental Methods**

**Isolation of nematophin**

*X. nematopbilus* YL001 was cultured for 72 h in a 70-L bioreactor (Eastbio, China) with 50.0 L medium consisting of the following components (g/L): glucose 6.13, peptone 21.29, MgSO_4_∙7H_2_O 1.50, (NH_4_)_2_SO_4_ 2.46, KH_2_PO_4_ 0.86, K_2_HPO_4_ 1.11 and Na_2_SO_4_ 1.72 at 28℃, pH 7.2, aeration ratio of 40 L/min and agitation speed of 150 rpm. The 50 L culture was centrifuged (12000 × g, 20 min, 4 °C) to separate the bacterial cells. The cell filtrate (50 L) was applied to a column of D101 resin (100 cm × 8 cm) at 500 mL min^-1^. After washing the column with H_2_O, MeOH was pumped onto the column and the eluate collected in 20-liter aliquots. The eluate was concentrated to dryness on a rotary evaporator, and lyophilized to yield crude product. The crude product (248 g) was re-dissolved in H_2_O (1000 mL), and was extracted with equal volume of ethyl acetate four times. The mixed extracts were dried with anhydrous sodium sulfate, and filtered, and the filtrate was concentrated on a rotary evaporator to yield brown oil. The crude oil (15.6 g) was purified by column chromatography on silica gel of 200 meshes.

**NMR analyses**

NMR experiments were performed in deuterated chloroform with a symmetrical NMR microtube susceptibility-matched with the appropriate solvent (Shigemi, Inc.) on a Brucker Avance 600 NMR. Nematophin were confirmed by ^1^H NMR and mass spectrometry. ^1^H and ^13^C data (Tables S3), MS data (Table S2), NMR spectra (Fig. S5, S6), and GC-MS spectra (Fig. S7) are shown for nematophin.

**GC-MS analysis of nematophin**

Analyse of nematophin were performed using a Focus gas chromatograph coupled to a Polaris Q ion trap mass spectrometer. Helium was used as carrier gas at a low rate of 1mL/min. A volume of 2.0 μL was injected. Separation of nematophin was performed on a TR-1MS capillary column (30 m × 0.25 mm i.d., 0.25 μm film thickness). Injector and ion source temperatures were 250∘C and 200∘C, respectively. A splitless injection mode was selected with a solvent delay time of 3 min. The GC conditions were the following: the initial temperature of the column was held at 160 °C for 1 min programmed at 3.0 °C min^–1^ to 250 °C, and at 250 °C for 5 min. Spectra were acquired in single ion monitoring (SIM) mode. MS detection was implemented with electron ionization (electron energy of 70 eV) and full scan mode (*m/z* 30–600).

**Quantiﬁcation analysis of nematophin via HPLC**

*X. nematophila* was cultured in 250-mL Erlenmeyer flasks containing 50 mL medium with varying initial pH (5.5, 7.0 and 8.5) at 30 ºC and 150 rpm. After 72 h incubation, 100 mL cultures were centrifuged (12000 × g, 20 min, 4 °C) to separate the bacterial cells and the cell filtrate were extracted with equal volume of ethyl acetate four times, mixed, and the water in the extracts was removed by anhydrous sodium sulfate. The ethyl acetate fractions were concentrated to dryness on a rotary evaporator and the residue was re-dissolved in MeOH (1.0 mL), and then ﬁltered through a 0.22 μm ﬁlter before HPLC analysis.

The HPLC system (Waters 600E, Milford, USA) was equipped with a Waters 2478 UV detector and a Thermo C_18_ column (250 mm × 4.6 mm). The gradient program was as follows: 0-2 min, 10% MeCN; 2-20 min, 10-40% MeCN; 20-35 min; 40-100% MeCN. The system was run at 25 ℃ at a ﬂow rate of 1 mL/min. The sample injections were 10 μL in volume. Nematophin was quantiﬁed using external standard method and the wavelength was set at 254 nm. Seven diluted concentrations of nematophin standard samples, 4000, 2000, 1000, 500, 250, 125 and 62.5 μg/mL were used and the established linear regression equation was *y*=2273.8*x*+188385, *R*^2^ = 0.9937. The precisions for all the concentrations were below 2 %.

**Table S1** Primers used in this study

| Primer | Sequence (5’-3’) | Size of fragment |
| --- | --- | --- |
|  |  |  |
|  |  |  |
| For RT-PCR analysis  *xcnA*-F | CTGTCTGAAGATAAGCAGAGCTGG | 674 |
| *xcnA*-R | GGGAATTCGAATACCTTCCCAGAC |
| *xcnB*-F | GTTCATATCGGATGCTGAAACCTG | 461 |
| *xcnB*-R | CCAGAAATGCTGCTTCGTTCATG |
| *xcnC*-F | AAGTGACTTGCATTGATGCGTTG | 55 |
| *xcnC*-R | ACATTTGTCGATTACGAGGTGTACG |
| *xcnE*-F | ACAGTGCATACCAGTTTAGTCGG | 471 |
| *xcnE-*R | CCAGGCAATGCTGTAACGAC |
| *xcnF*-F | GGAGCCGGTAATCAGTGGTCATGG | 320 |
| *xcnF*-R | CTCATCAAATACCCGTGTCTTGCCGTC |
| *xcnG*-F | TCTTGCCAGAGCTCTCATTCTG | 524 |
| *xcnG*-R | AGTGTTAGGCTCCAGAATACGTTTC |
| *xcnH*-F | GAATAAACAGTTCGCACCTACTCTG | 593 |
| *xcnH*-R | GATTCACCAAGATACTCTGAAGCC |
| *xcnI*-F | AGTTCATTGCGATAGAGCTTCCTG | 327 |
| *xcnI*-R | TCAGTGAGTTCTTTGGTATCACCC |
| *xcnK*-F | CATCCTTTGCCTCTCAGTTGTTAAG | 540 |
| *xcnK*-R | GGTACTATATGGAGGGTATGACCG |
| *xcnL*-F | CTAAATCCATTCTCGGGCATCTTG | 565 |
| *xcnL*-R | CATACCCGGATATTGTGTTCCTTG |
| *xcnM*-F | AATCAGTACGCTCCTCATCTGG | 327 |
| *xcnM*-R | AACAACACTTGCCATCCATGAAG |
| *xcnN*-F | GATAGGTTCAACACAACGAGCATT | 422 |
| *xcnN*-R | CTATTGACTCATTGTTATCTCCAGCC |
| *isnA*-F | CTGAGGATGTATTGAAAGACGGAT | 613 |
| *isnA*-R | GCAAGGAAAACCGATAGAATGT |
| *isnB*-F | AAATAAACGCTTCACGACCA | 734 |
| *isnB*-R | GAAACCTTTTGGGCTGCTTA |
| *isnC*-F(glycosyltransferase) | ACCCCGTTGTCGTATTCA | 520 |
| *isnC*-R(glycosyltransferase) | CCACCACTCTGGCATTAT |
| 16*S* rDNA-F | GGTAGTAAATGTTGGGGGATTTTCCC | 715 |
| 16*S* rDNA-R | GACATCGTTTACAGCGTGGACTAC |
| For qRT-PCR analysis  *isnA*-F | CCCGATACAACAACAACCCTTCTT | 126 |
| *isnA-*R | CGCTCACGCAATATACCGATGAT |
| *isnB*-F | CTCTTGGTGCATCGGTTCGTTGT | 88 |
| *isnB*-R | TGGTGGTGAAGTTGTTTCGCCATT |
| *isnC*-F(glycosyltransferase) | CGTATTCCTGTGGTCGCTATTGGT | 146 |
| *isnC*-R(glycosyltransferase) | ATCAACTGCTGGGTTTCCTCATCC |
| *ompR*-F | GAGCAGGGATTTCAAGTTCGTAGTG | 137 |
| *ompR*-R | CTCAATCTTCGGCAGACAGACAGT |
| *cpxR*-F | CCGTCAAAACTGGCCTCC | 171 |
| *cpxR*-R | CGCAGATGACTATCTCCCTAAACC |
| *recA-*F | TGATGAAGTTGTTGGTAGCGAAACG | 134 |
| *recA*-R | ACTCAGATCGATCAACTCTCCCAG |

**Table S2** Information on the identified metabolites in ethyl acetate extract of *X. nematophila* YL001 based on LC-MS/MS

| Chemical Name | Elemental  composition | Theoretical  (m/z) | Measured  (m/z) |
| --- | --- | --- | --- |
| Nematophin 1 | C_16_H_20_N_2_O_2_ | 273.15 | 273.15 |
| Indole derivatives 2 | C_15_H_18_N_2_O_2_ | 259.14 | 259.14 |
| Indole derivatives 3 | C_16_H_22_N_2_O_2_ | 275.17 | 275.16 |
| Indole derivatives 4 | C_15_H_20_N_2_O_2_ | 261.15 | 261.12 |
| Rhabduscin 5 | C_17_H_20_N_2_O_5_ | 333.14 | 333.09 |

**Table S3** ^1^H and ^13^C data for nematophin in CDCl_3_. Chemical shifts (δ) in ppm

| Atom | **δ_H_**  (mult, J [Hz]) | **δ_C_** |
| --- | --- | --- |
| NH | 8.03 (l H, bs) |  |
| 1-NH | 7.11(1H, bd, *J*=3.0 Hz) |  |
| 2 | 7.07(1H, bd, *J*=3.0 Hz) | 112.3 |
| 3 |  | 112.6 |
| 4 | 7.66(1H, dd, *J*=9.8Hz,0.75 Hz) | 118.7 |
| 5 | 7.27(1H, td, *J*=10.1Hz,1.5 Hz) | 122.0 |
| 6 | 7.18(1H, td, *J*=9.9Hz,1.3Hz) | 119.6 |
| 7 | 7.44(1H, d, *J*=10.0 Hz) | 111.3 |
| 8 |  | 127.2 |
| 9 |  | 136.5 |
| 10 | 3.07(2H, t, *J*=5.1 Hz) | 25.5 |
| 11 | 3.70(2H, q, *J*=5.0 Hz) | 39.6 |
| 1’ |  | 202.4 |
| 2’ |  | 160.1 |
| 3’ | 3.55(1H, sext, *J*=4.9 Hz) | 40.4 |
| 4’ | 1.79(1H, m) | 25.2 |
|  | 1.46(1H, m) |  |
| 5’ |  | 11.5 |
| 3’-CH_3_ |  | 15.2 |

**
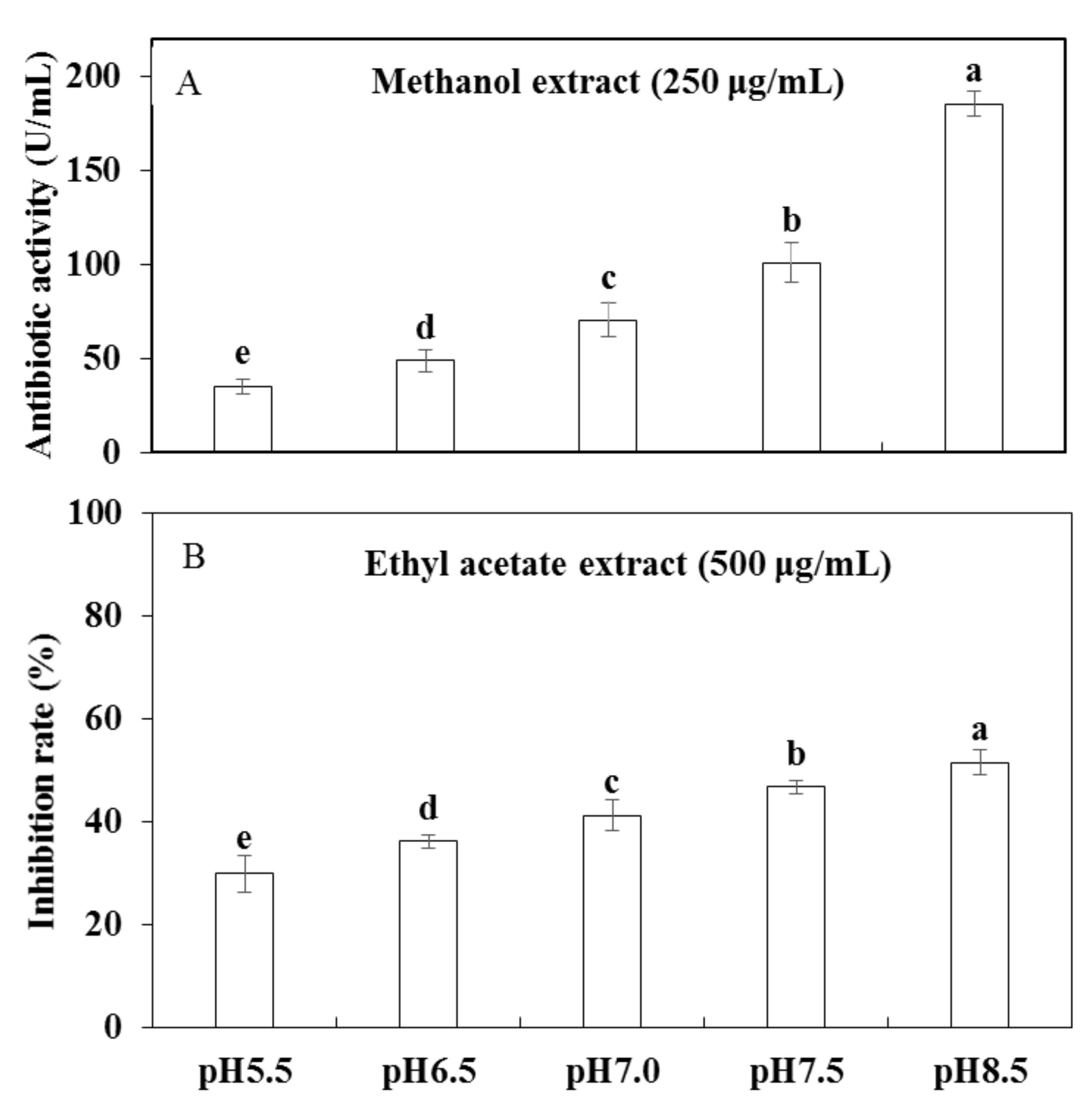
**

**Fig. S1** Inhibitory effect of ethyl acetate extract and methanol extract of the cell-free filtrate of *X. neamatophila* YL001 at different initial pH on *B. subtilis* (A) and *B. cinerea* (B). The inhibitory effect of *X. nematophila* YL001 culture on *B. subtilis* is presented as antibiotic activity which was expressed as units of activity per mL of the cell-free filtrate generated as described in *Materials and Methods*. The inhibitory effect of *X. nematophila* YL001 culture on *B. cinerea* is presented as inhibition rate which was calculated described in *Materials and Methods*. Data are presented as the averages ± the standard deviations for six replicates. Different lower case letters above the bars indicate significant differences at *P* = 0.05.

**
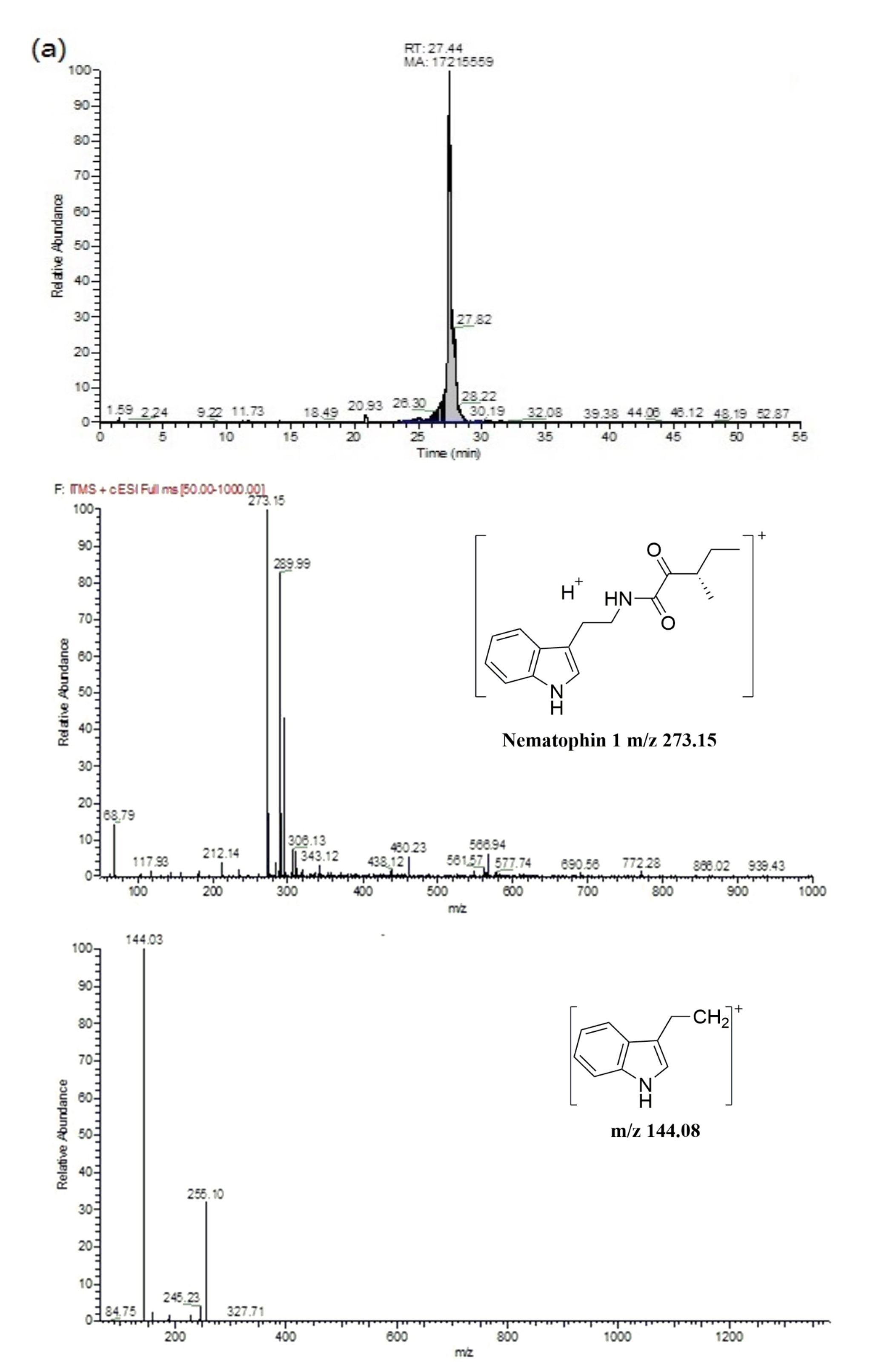
**


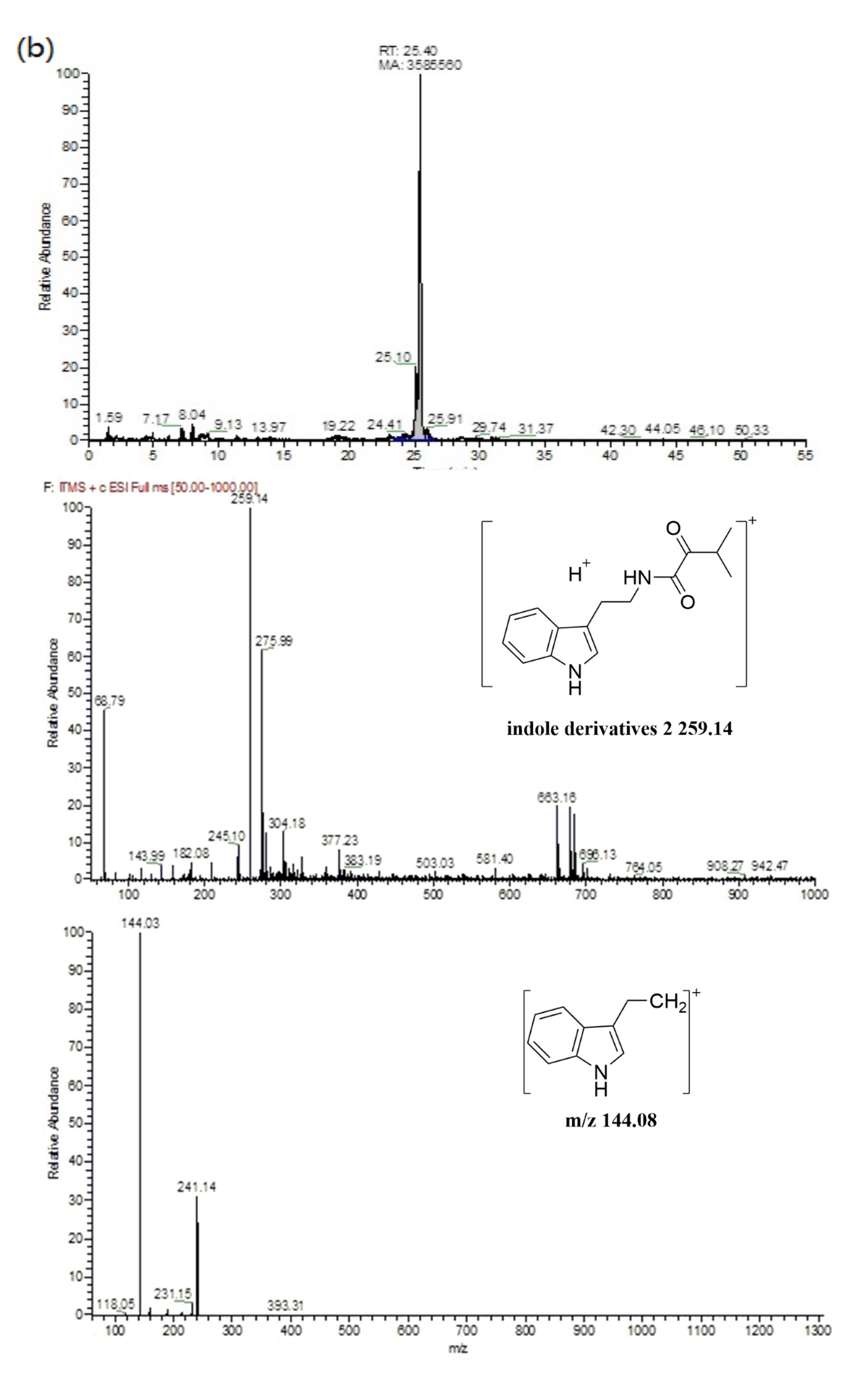

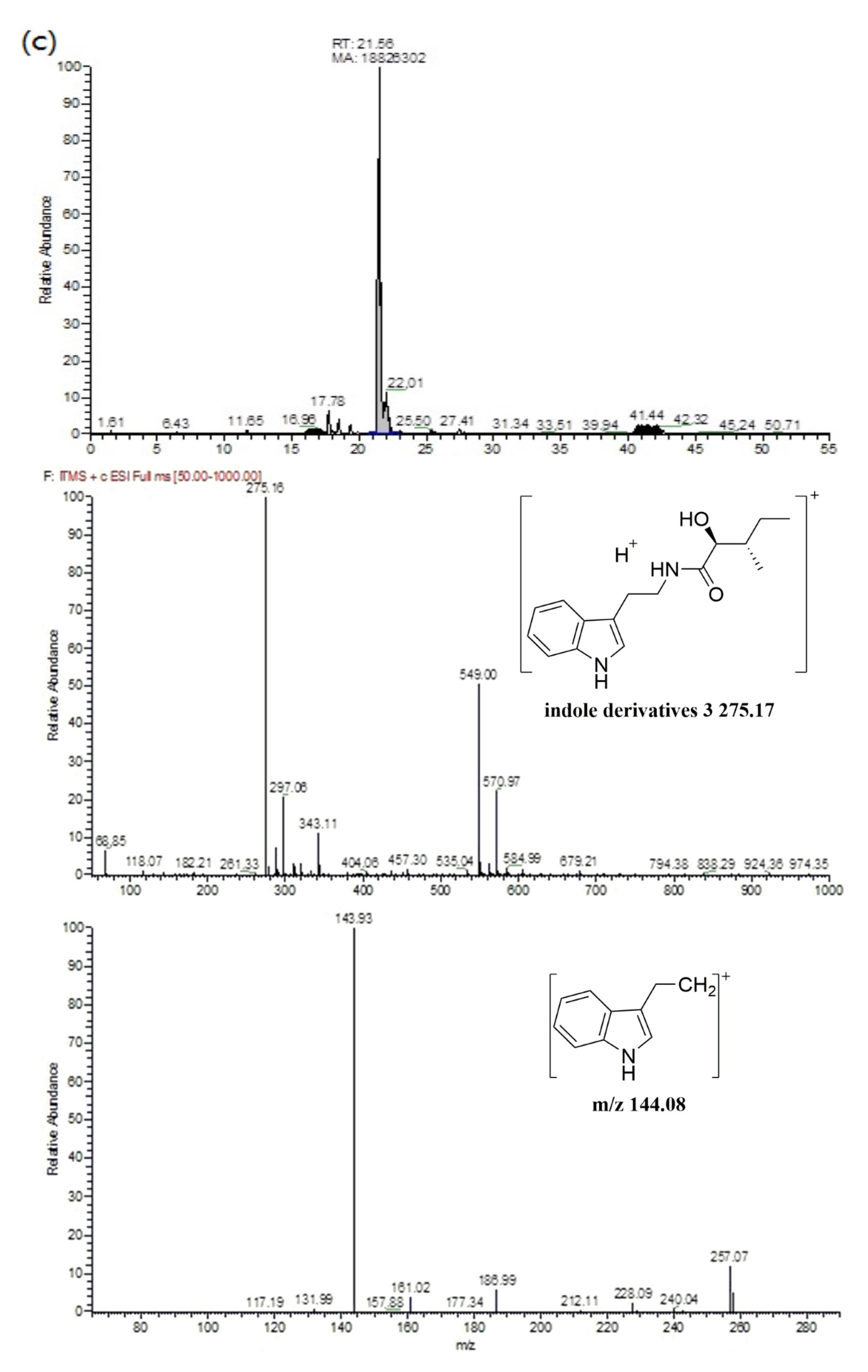


**
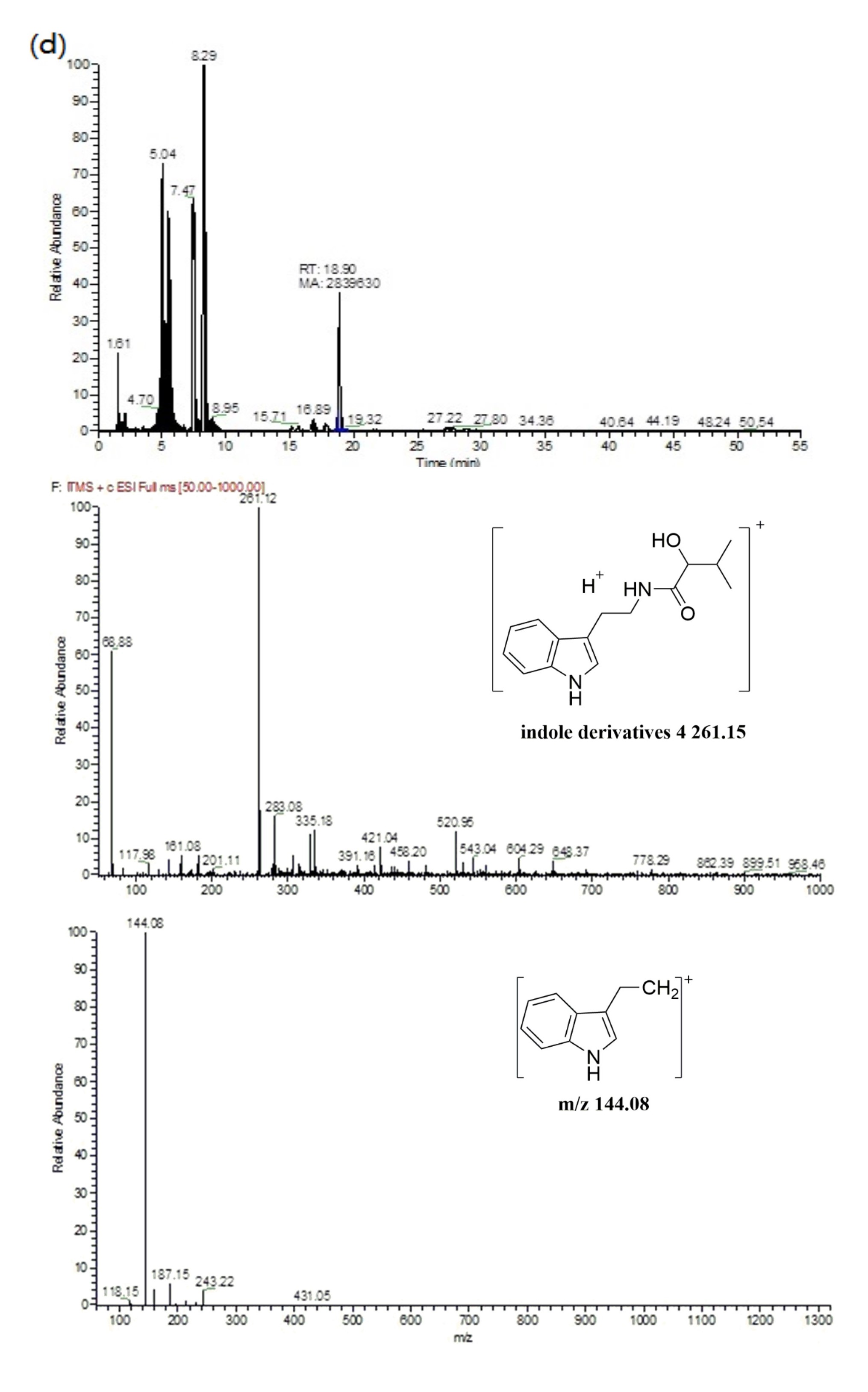

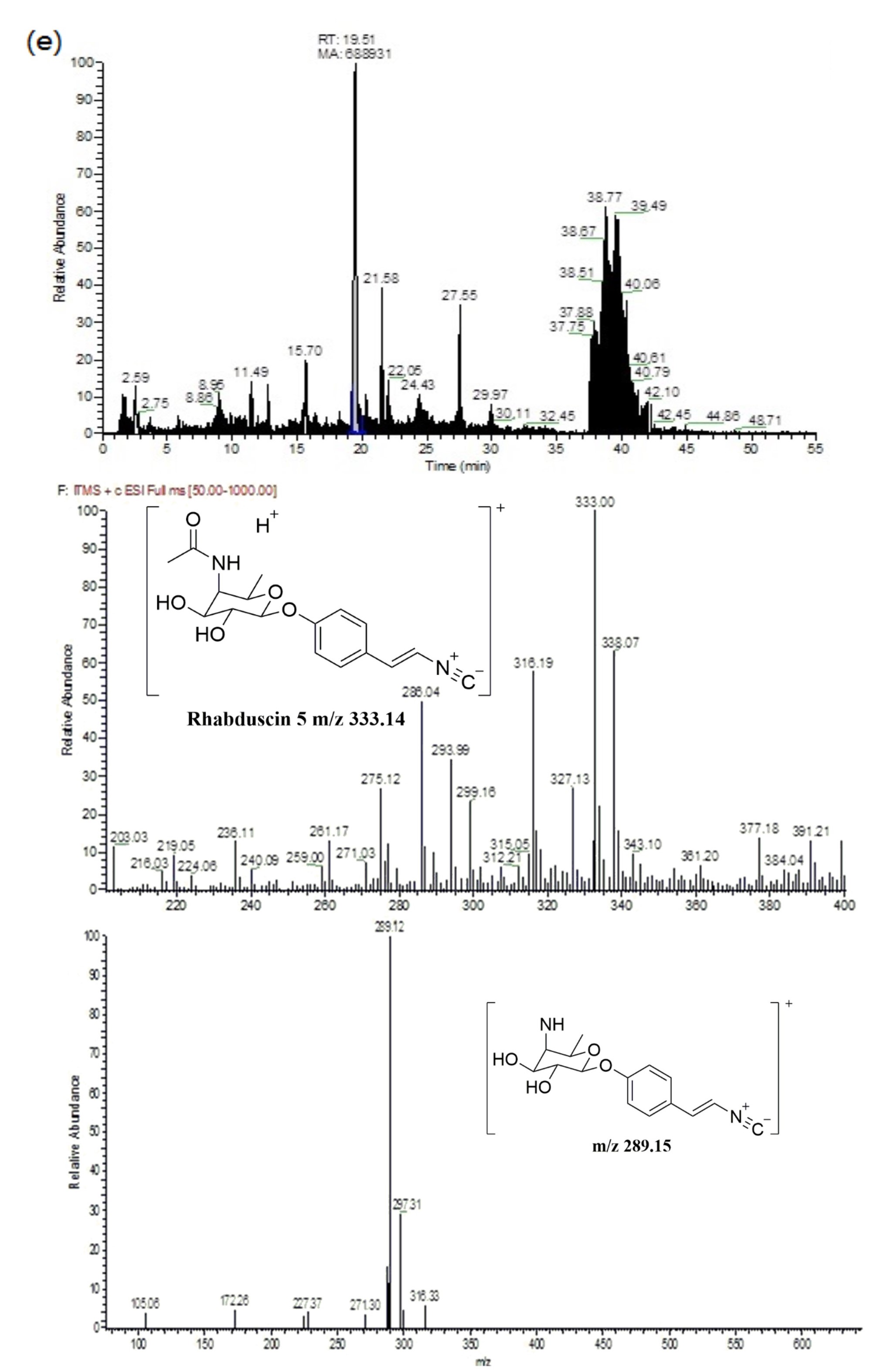
**

**Fig. S2** HPLC-MS TIC of the identified metabolites in ethyl acetate extract of *X. nematophila* YL001 at pH 8.5. (a) Extracted ion chromatography spectra of Nematophin 1 (UP), MS spectra (Middle) of Nematophin 1, [M+H]^+^=273.15; MS/MS spectra (Down) of Nematophin 1, [M+H]^+^=144.08 ; (b) Extracted ion chromatography spectra of Indole derivatives 2 (UP), MS spectra (Middle) of Indole derivatives 2, [M+H]^+^=259.14; MS/MS spectra (Down) of Indole derivatives 2, [M+H]^+^=144.08; (c) Extracted ion chromatography spectra of Indole derivatives 3 (UP), MS spectra (Middle) of Indole derivatives 3, [M+H]^+^=275.17; MS/MS spectra (Down) of Indole derivatives 3, [M+H]^+^=144.08; (d) Extracted ion chromatography spectra of Indole derivatives 4 (UP), MS spectra (Middle) of Indole derivatives 4, [M+H]^+^=261.15; MS/MS spectra (Down) of Indole derivatives 4, [M+H]^+^=144.08; (e) Extracted ion chromatography spectra of Rhabduscin 5 (UP), MS spectra (Middle) of Rhabduscin 5, [M+H]^+^=333.14; MS/MS spectra (Down) of Rhabduscin 5, [M+H]^+^=289.15.


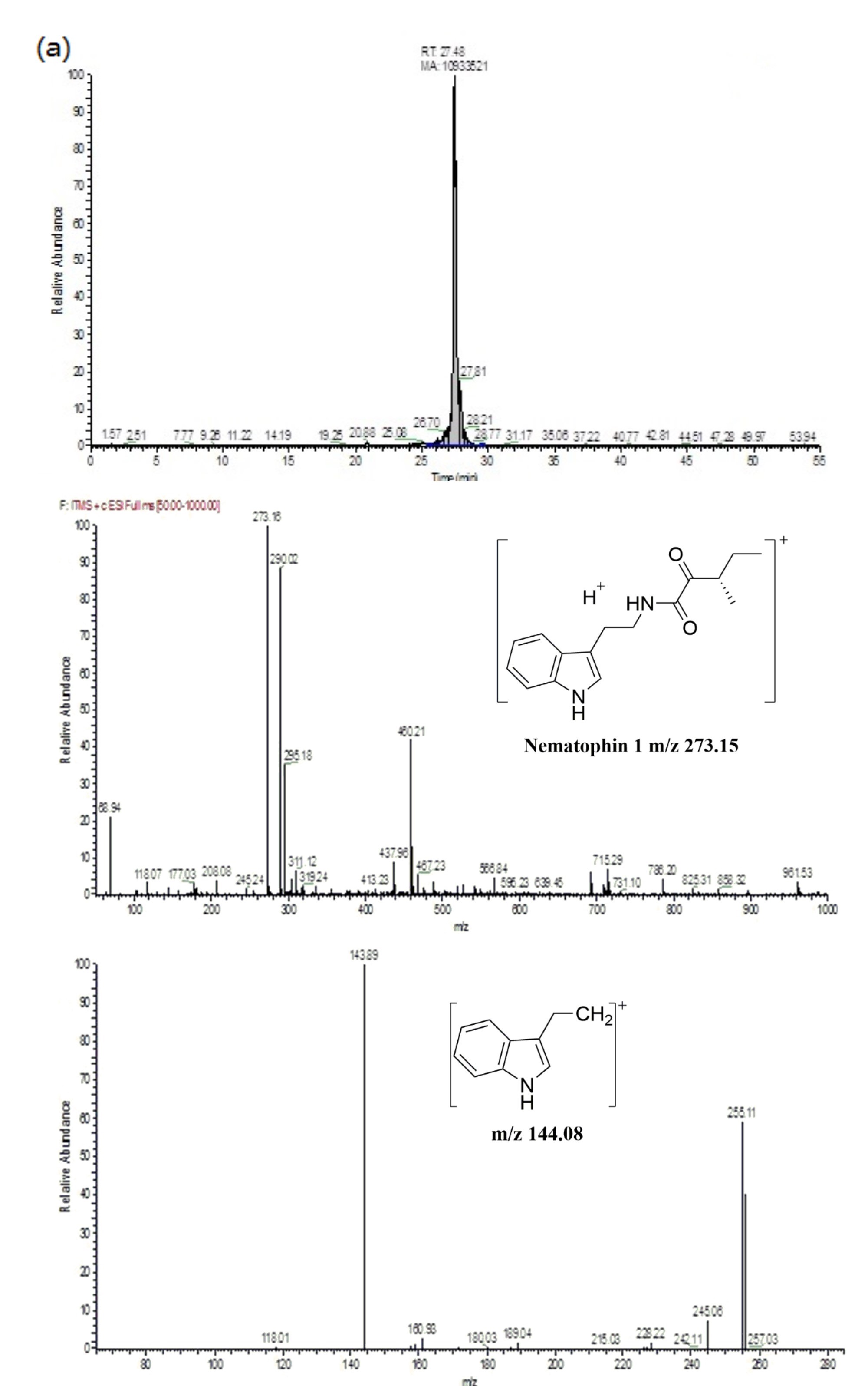


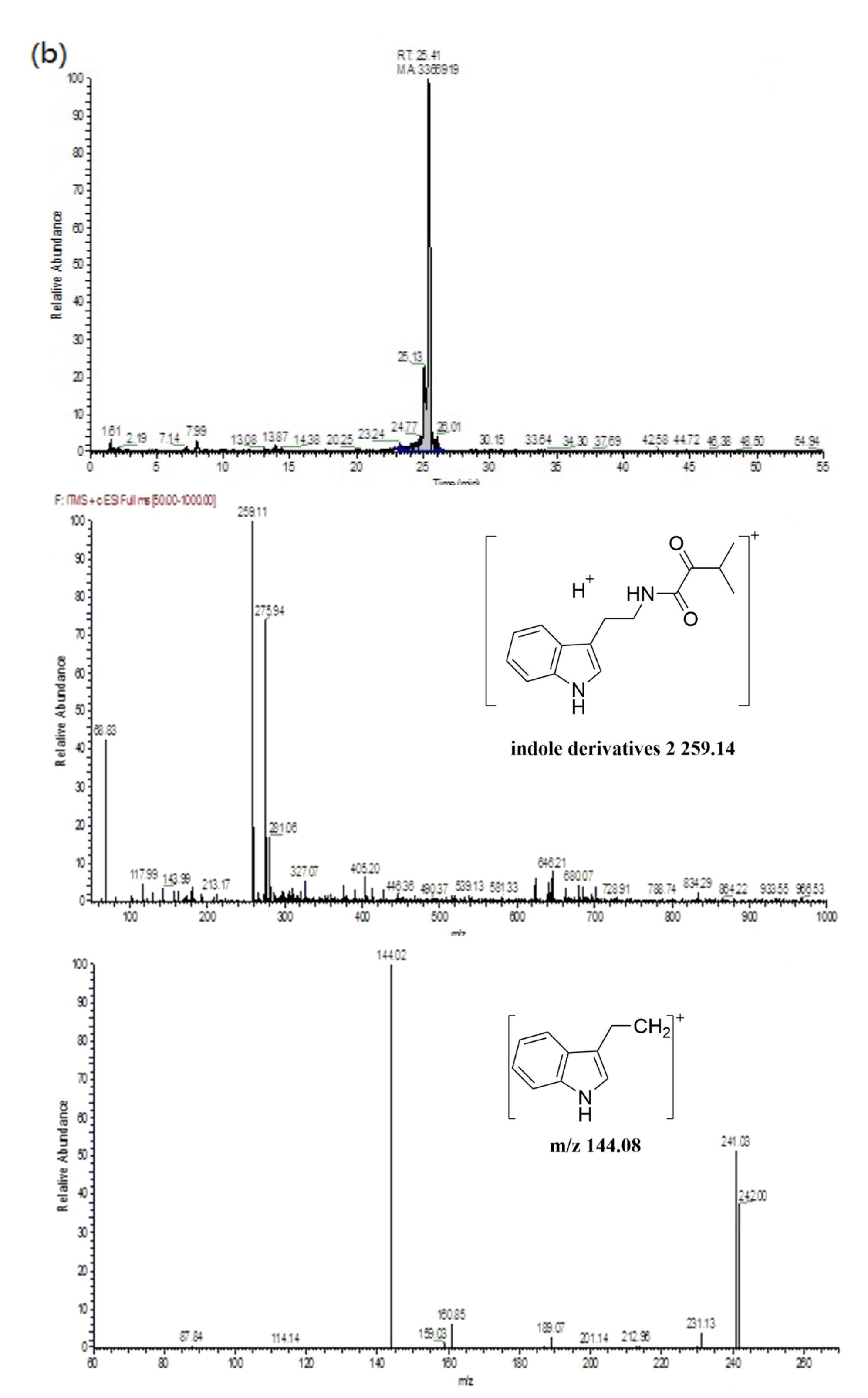


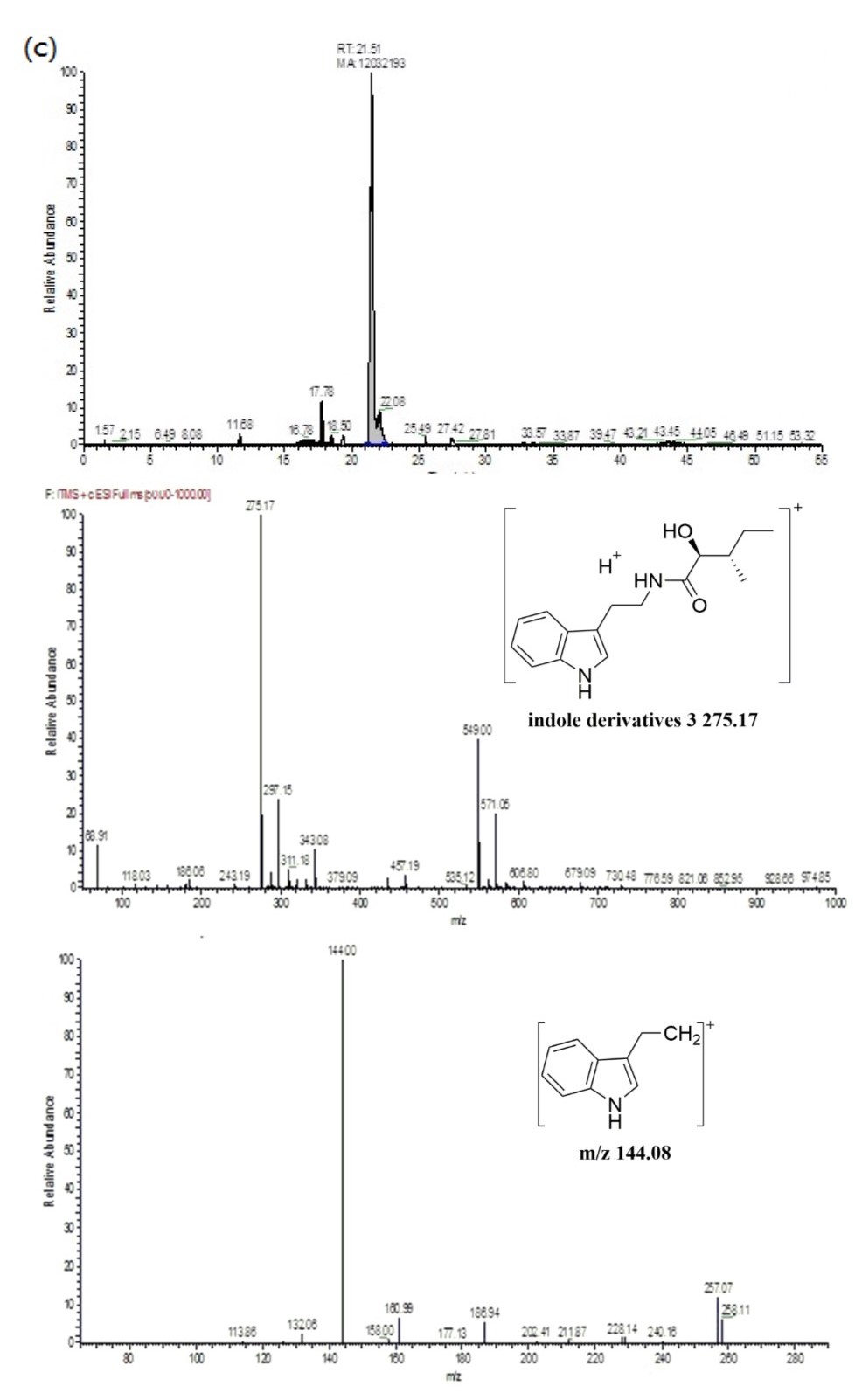


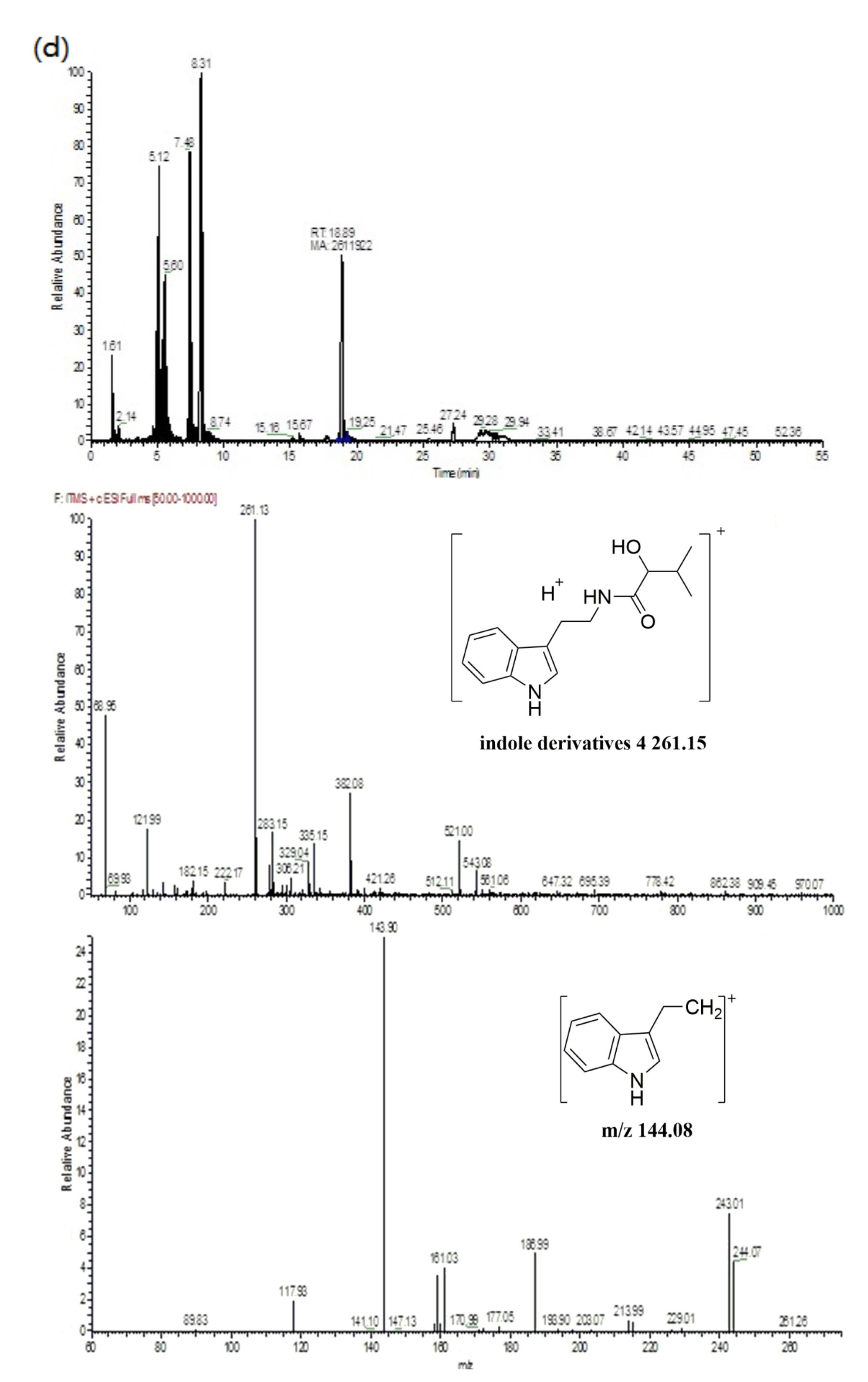


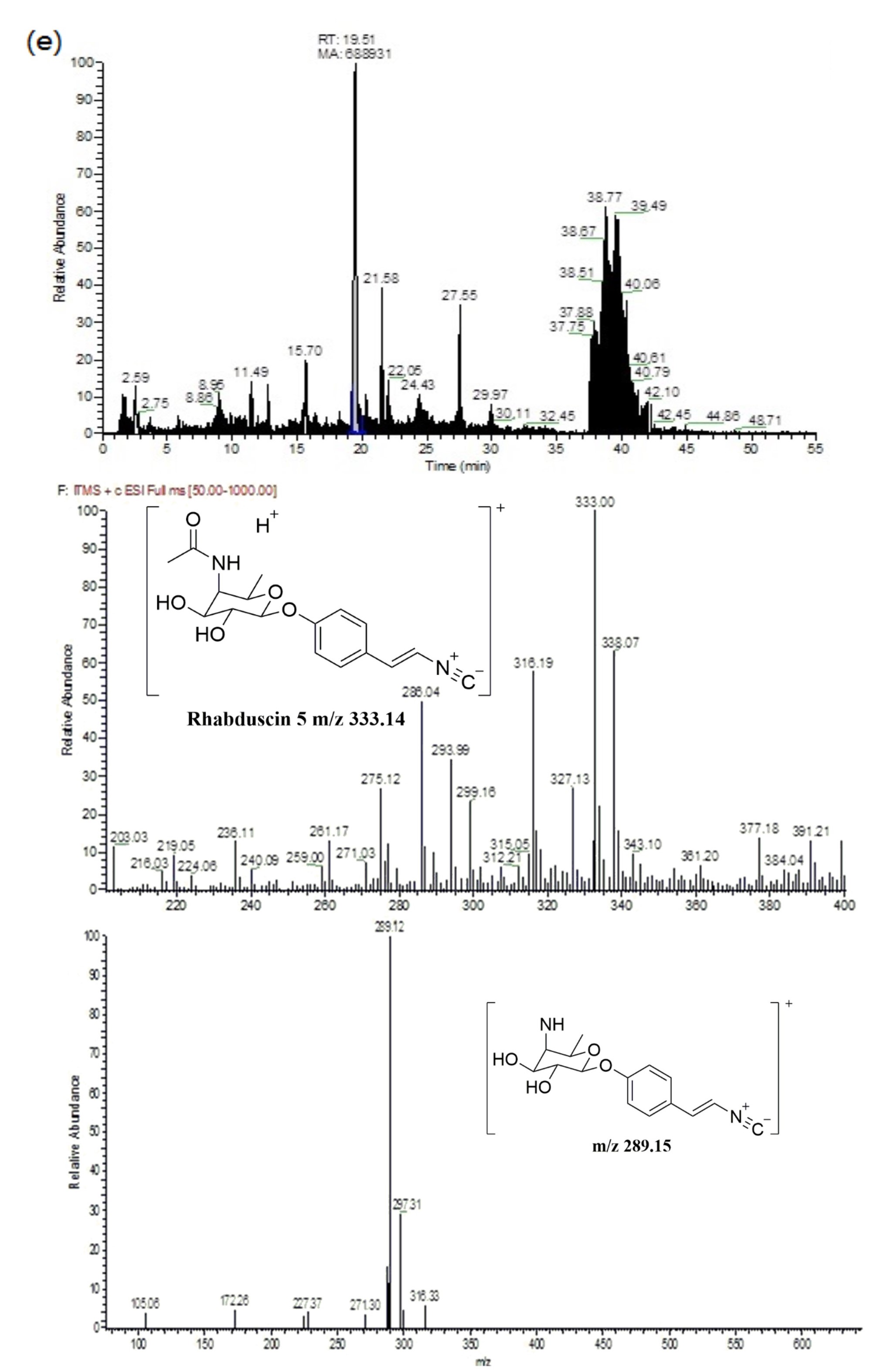


**Fig. S3** HPLC-MS TIC of the identified metabolites in ethyl acetate extract of *X. nematophila* YL001 at pH 7.0. (a) Extracted ion chromatography spectra of Nematophin 1 (UP), MS spectra (Middle) of Nematophin 1, [M+H]^+^=273.15; MS/MS spectra (Down) of Nematophin 1, [M+H]^+^=144.08 ; (b) Extracted ion chromatography spectra of Indole derivatives 2 (UP), MS spectra (Middle) of Indole derivatives 2, [M+H]^+^=259.14; MS/MS spectra (Down) of Indole derivatives 2, [M+H]^+^=144.08; (c) Extracted ion chromatography spectra of Indole derivatives 3 (UP), MS spectra (Middle) of Indole derivatives 3, [M+H]^+^=275.17; MS/MS spectra (Down) of Indole derivatives 3, [M+H]^+^=144.08; (d) Extracted ion chromatography spectra of Indole derivatives 4 (UP), MS spectra (Middle) of Indole derivatives 4, [M+H]^+^=261.15; MS/MS spectra (Down) of Indole derivatives 4, [M+H]^+^=144.08; (e) Extracted ion chromatography spectra of Rhabduscin 5 (UP), MS spectra (Middle) of Rhabduscin 5, [M+H]^+^=333.14; MS/MS spectra (Down) of Rhabduscin 5, [M+H]^+^=289.15.


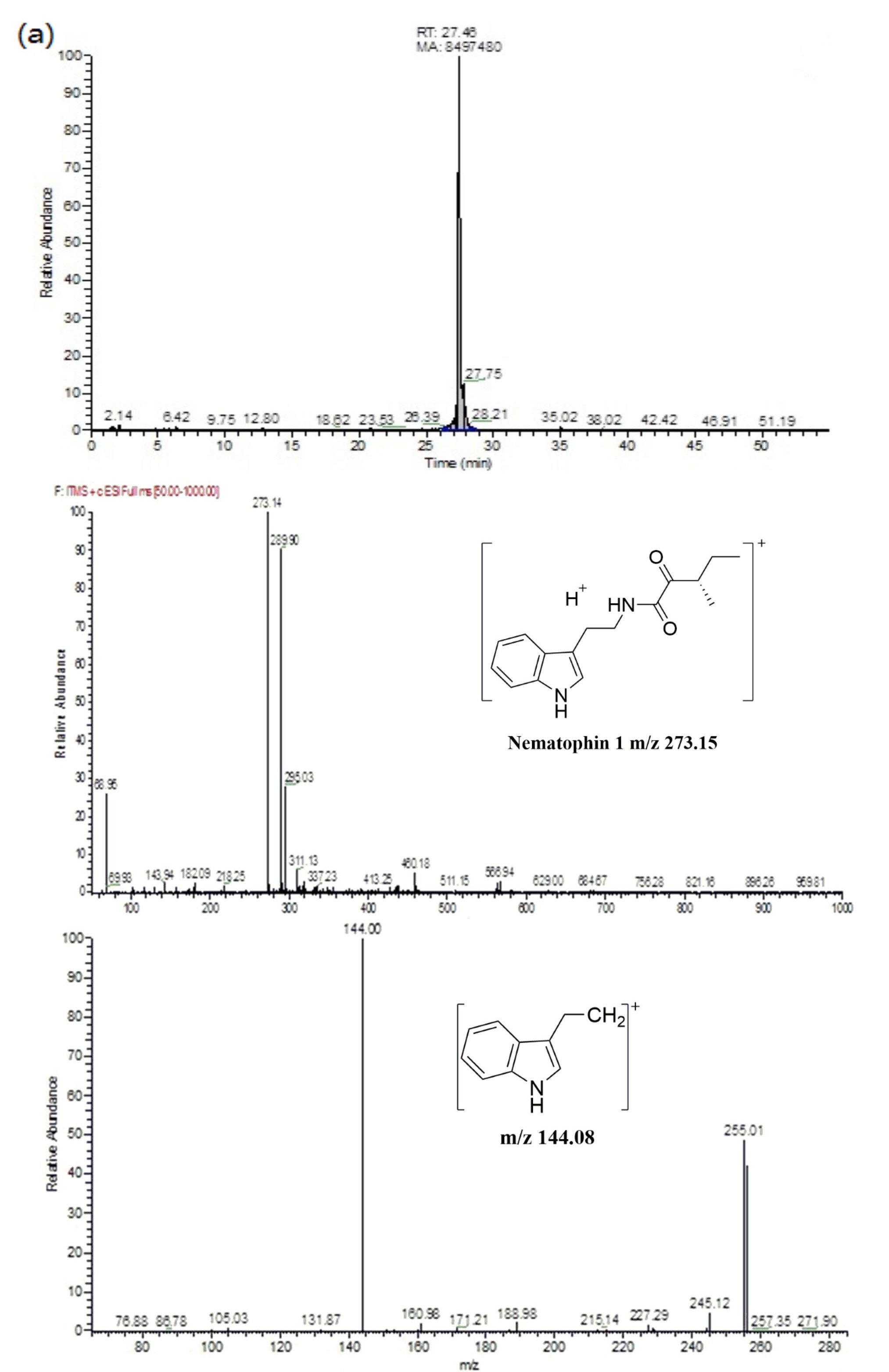


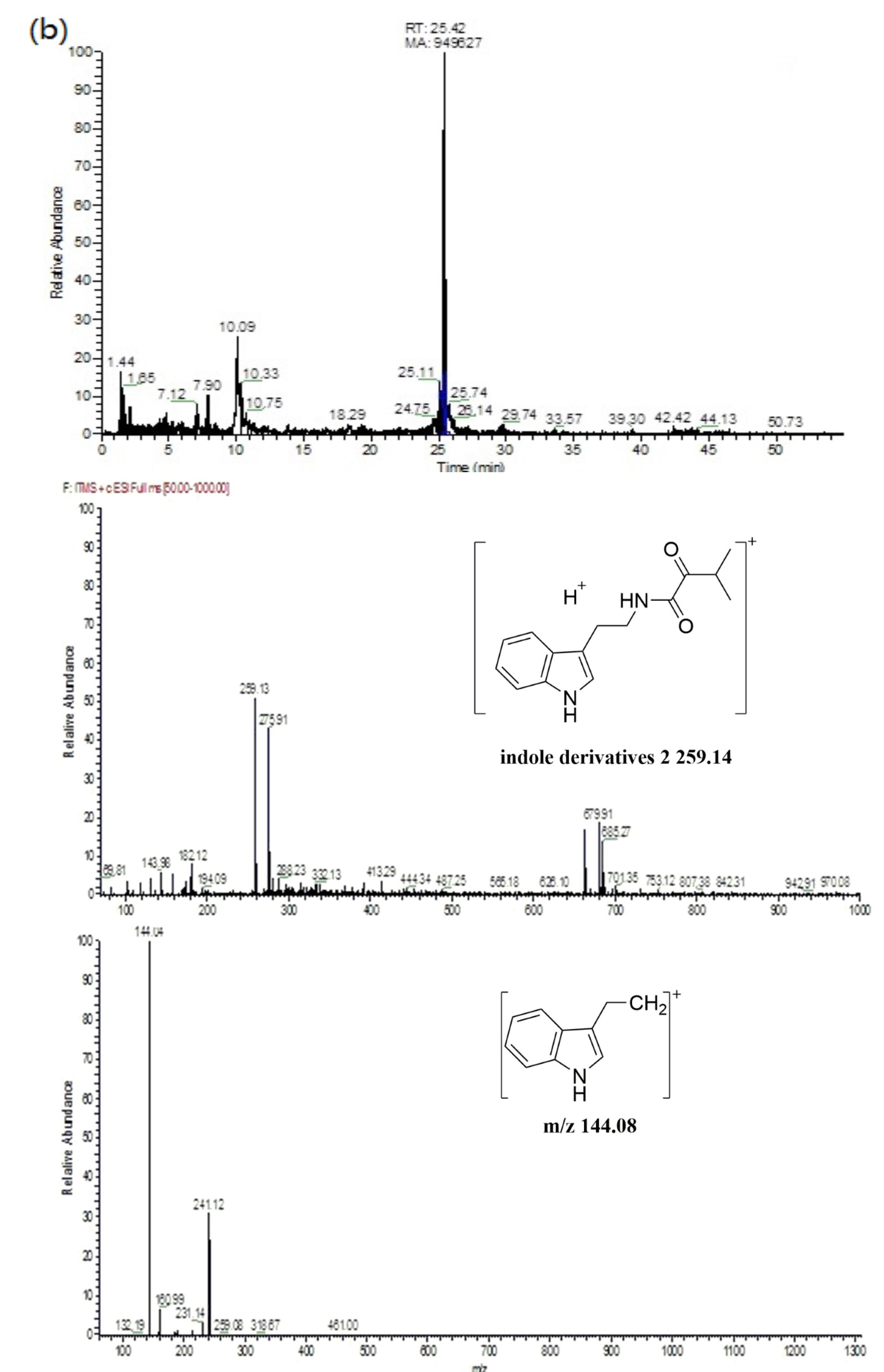


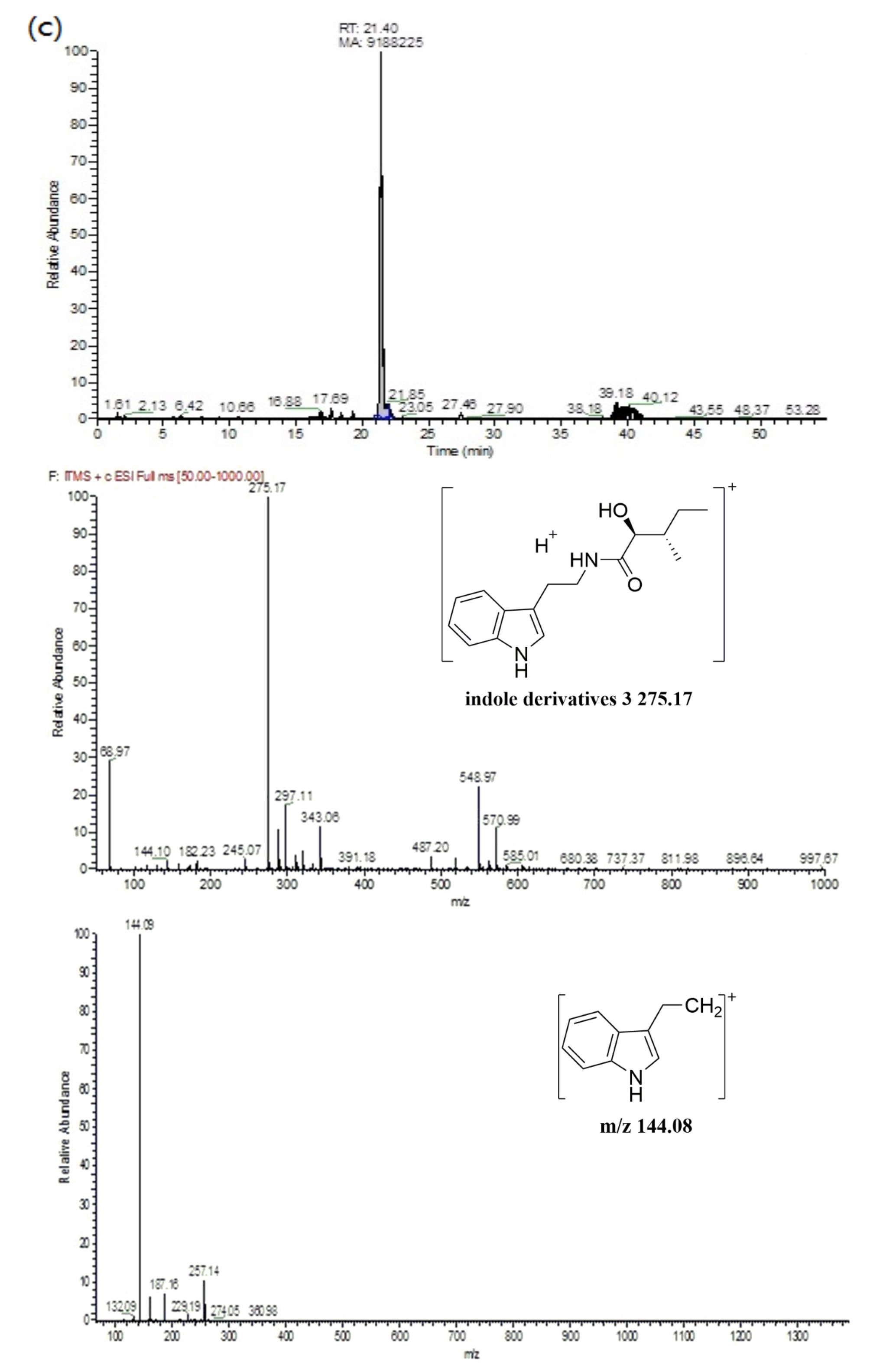

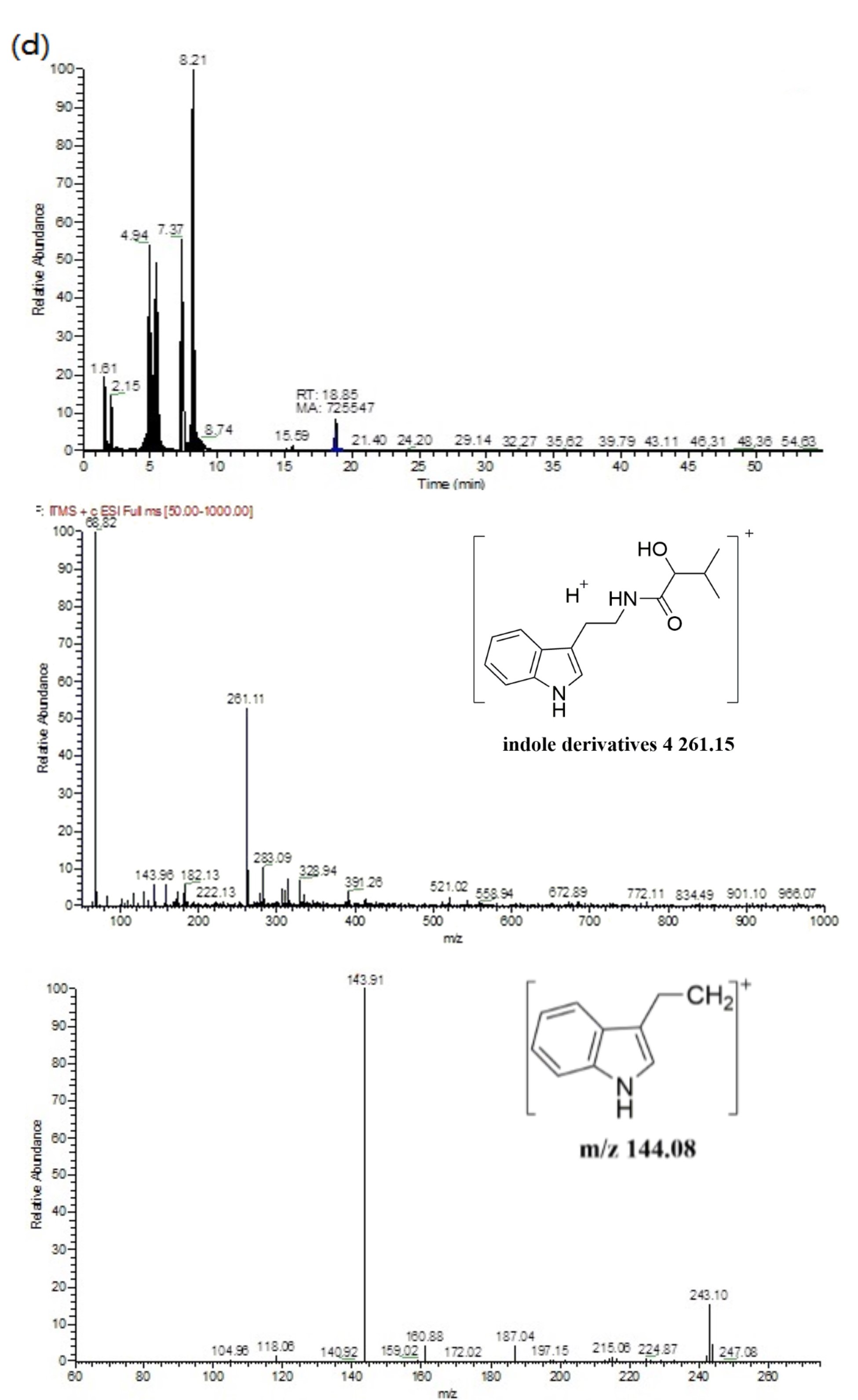

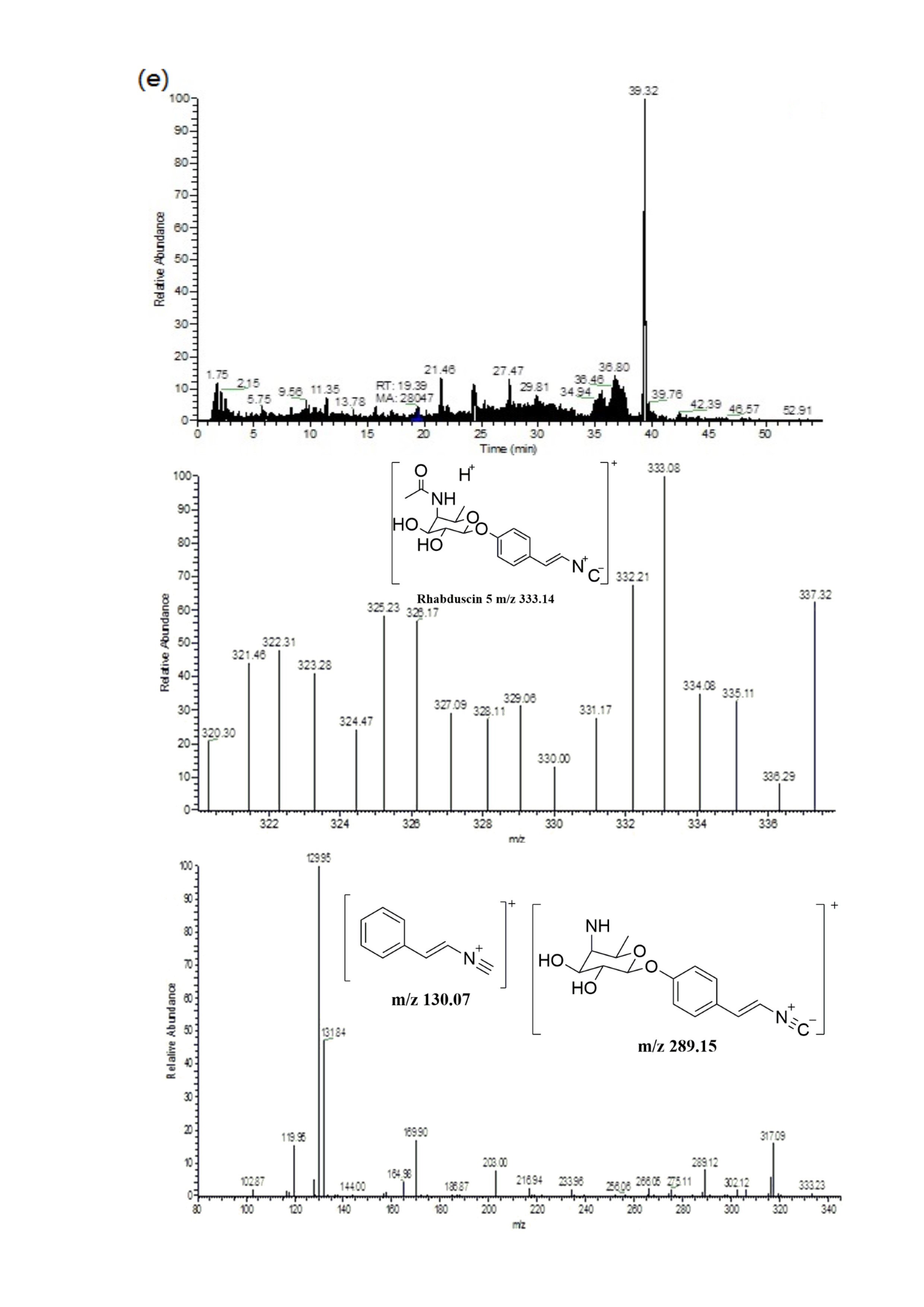


**Fig. S4** HPLC-MS TIC of the identified metabolites in ethyl acetate extract of *X. nematophila* YL001 at pH 5.5. (a) Extracted ion chromatography spectra of Nematophin 1 (UP), MS spectra (Middle) of Nematophin 1, [M+H]^+^=273.15; MS/MS spectra (Down) of Nematophin 1, [M+H]^+^=144.08 ; (b) Extracted ion chromatography spectra of Indole derivatives 2 (UP), MS spectra (Middle) of Indole derivatives 2, [M+H]^+^=259.14; MS/MS spectra (Down) of Indole derivatives 2, [M+H]^+^=144.08; (c) Extracted ion chromatography spectra of Indole derivatives 3 (UP), MS spectra (Middle) of Indole derivatives 3, [M+H]^+^=275.17; MS/MS spectra (Down) of Indole derivatives 3, [M+H]^+^=144.08; (d) Extracted ion chromatography spectra of Indole derivatives 4 (UP), MS spectra (Middle) of Indole derivatives 4, [M+H]^+^=261.15; MS/MS spectra (Down) of Indole derivatives 4, [M+H]^+^=144.08; (e) Extracted ion chromatography spectra of Rhabduscin 5 (UP), MS spectra (Middle) of Rhabduscin 5, [M+H]^+^=333.14; MS/MS spectra (Down) of Rhabduscin 5, [M+H]^+^=289.15.


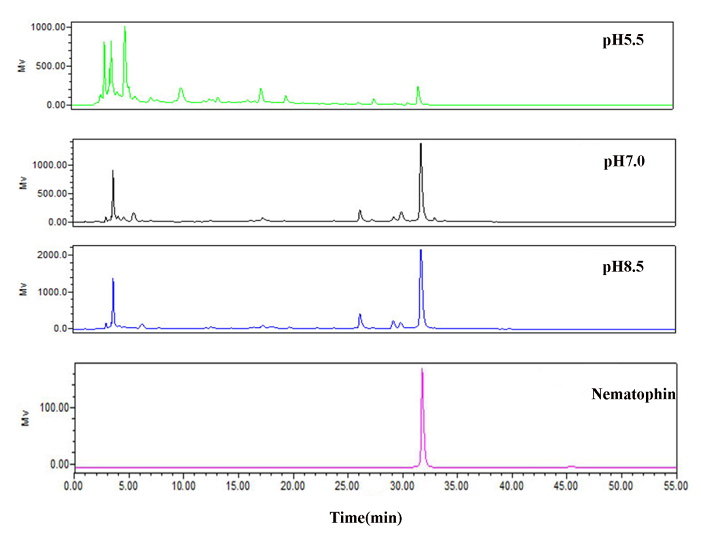


**Fig. S5** HPLC analysis of nematophin produced by *X. nematophila* YL001 at pH 5.5, 7.0 and 8.5. Registrations of peak and retention times are recorded through UV detection at 254 nm. Sample at different pH showed a peak, with a retention time (33.9 min) identical to that of standard nematophin. The concentration of standard nematophin was 250 μg/mL. The ethanol extract of 100 mL cultures was dissolved in 1.0 mL MeOH. The sample injections were 10 μL in volume.

**­­Fig. S6** ^1^H-NMR spectra (500 MHz，CDCl_3_) of nematophin

**Fig. S7** ^13^C -NMR spectra (125 MHz，CDCl_3_) of nematophin

**Fig. S8** GC-MS analysis of nematophin. (Up) GC chromatogram of nematophin. (Down) MS fragmentation profile of nematophin, the molecular ion *m/z* 272.04 [M+H]^+^, the fragment ion *m/z* 144.16 [M+H]^+^, *m/z* 143.22 [M+H]^+^, *m/z* 1130.27 [M+H]*^+^*, *m/z* 103.15 [M+H]^+^ and *m/z* 77.56 [M+H]^+^. MS detection was implemented with electron ionization (electron energy of 70 eV) and full scan mode (*m/z* 30–600). The concentration of nematophin was 250 μg/mL. The sample injections were 2.0 μL in volume.


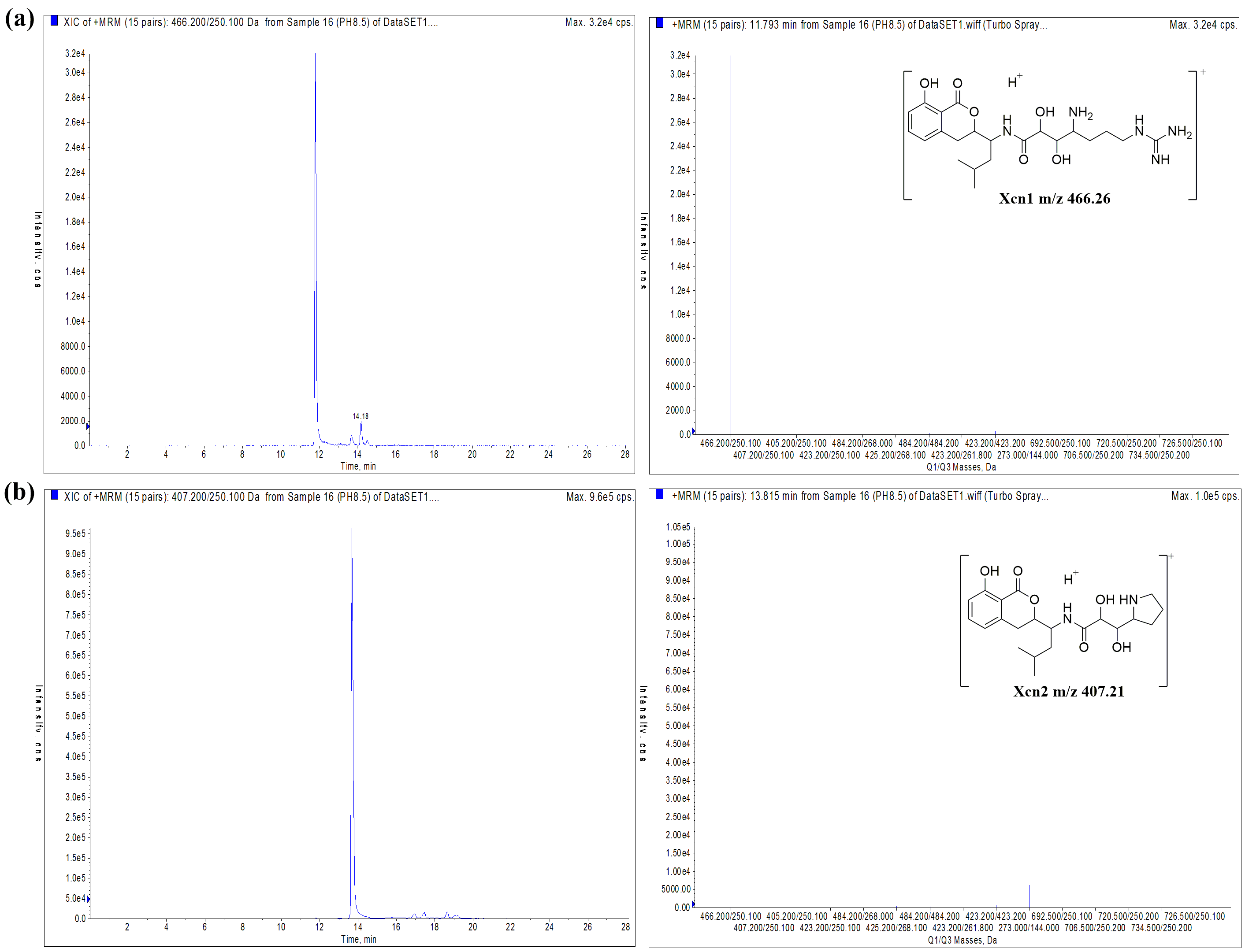


**Fig. S9** HPLC-MS analysis of Xcn1 and Xcn2 produced by *X. nematophila* YL001 at pH 8.5. (a): extracted ion chromatogram (EIC) of Xcn1 (left), the molecular ion peaks of Xcn1 ([M+H]^+^, 466 m/z)) (right). (b): extracted ion chromatogram (EIC) of Xcn2 (left), the molecular ion peaks of Xcn2 ([M+H]^+^, 407 m/z)) (right).


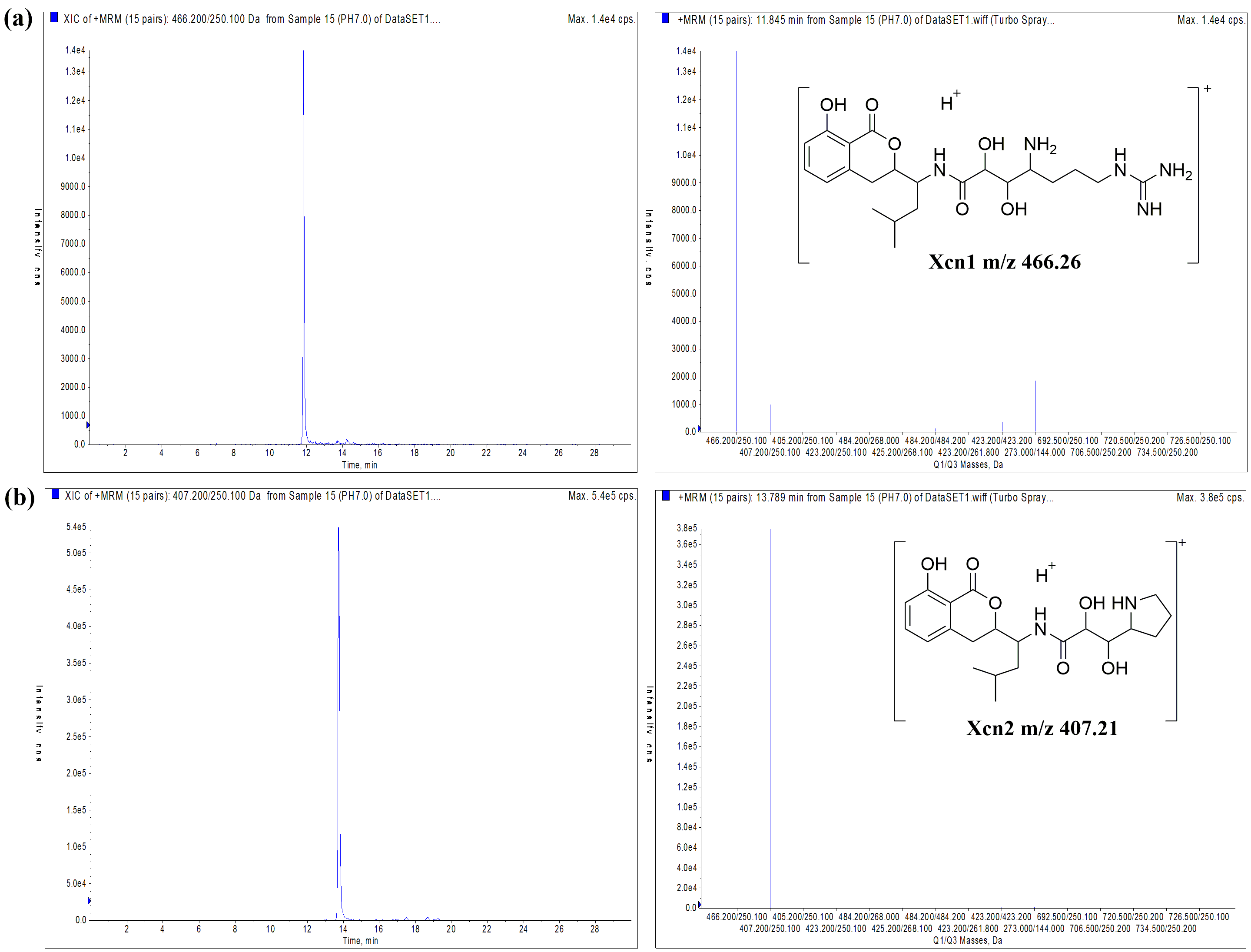


**Fig. S10** HPLC-MS analysis of Xcn1 and Xcn2 produced by *X. nematophila* YL001 at pH 7.0. (a): extracted ion chromatogram (EIC) of Xcn1 (left), the molecular ion peaks of Xcn1 ([M+H]^+^, 466 m/z)) (right). (b): extracted ion chromatogram (EIC) of Xcn2 (left), the molecular ion peaks of Xcn2 ([M+H]^+^, 407 m/z)) (right).

**
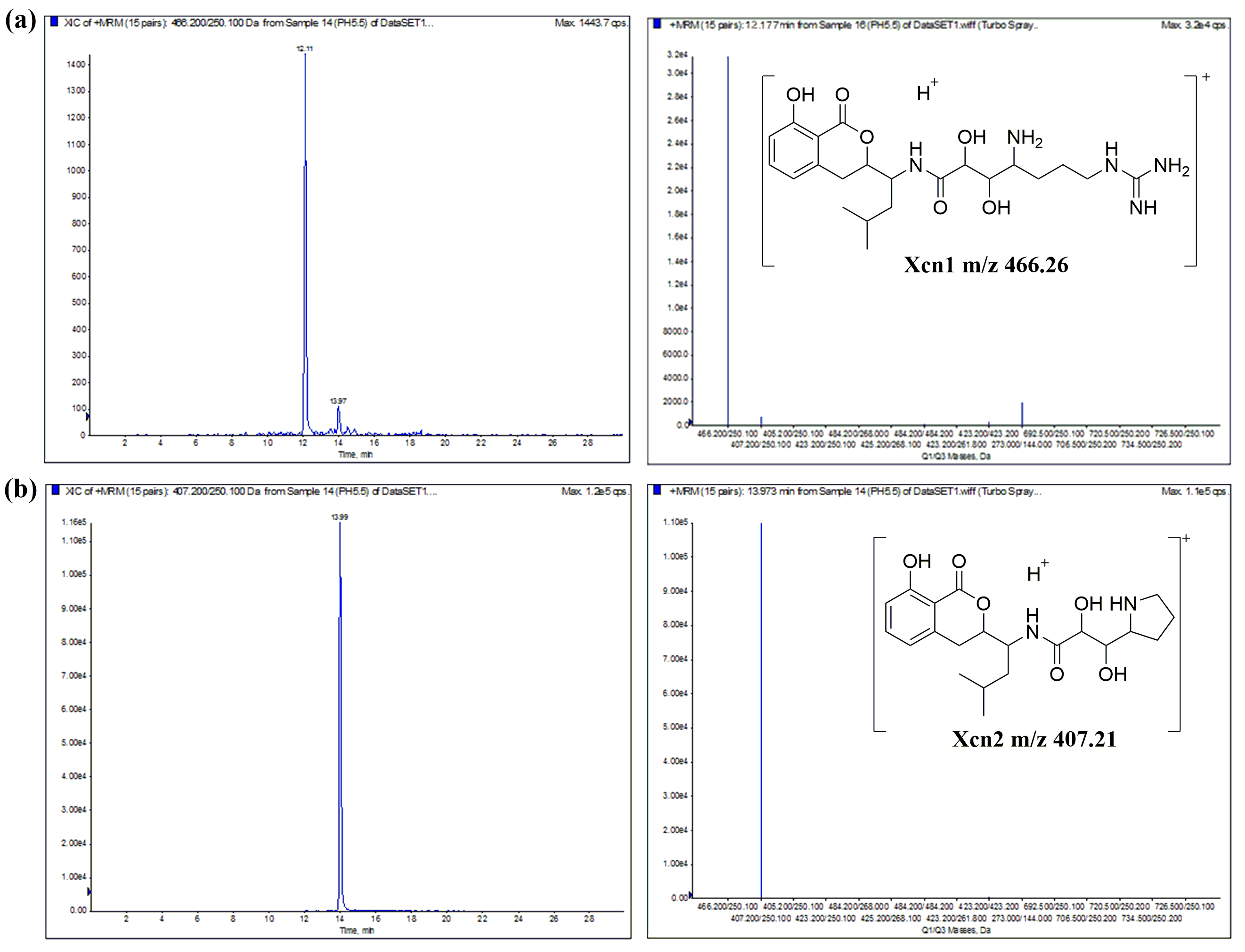
**

**Fig. S11** HPLC-MS analysis of Xcn1 and Xcn2 produced by *X. nematophila* YL001 at pH 5.5. (a): extracted ion chromatogram (EIC) of Xcn1 (left), the molecular ion peaks of Xcn1 ([M+H]^+^, 466 m/z)) (right). (b): extracted ion chromatogram (EIC) of Xcn2 (left), the molecular ion peaks of Xcn2 ([M+H]^+^, 407 m/z)) (right).
